# 3D Customized Silica‐Based AFM Probes Fabricated by Selective Laser Etching

**DOI:** 10.1002/smtd.202501772

**Published:** 2026-01-19

**Authors:** Koutayba Saada, David Bourrier, Juliette Lignieres, Etienne Dague, Laurent Malaquin

**Affiliations:** ^1^ LAAS‐CNRS Université de Toulouse CNRS Toulouse 31400 France

**Keywords:** 3D glass microsystems, AFM, AFM probes, cantilever, FLICE, selective laser etching (SLE)

## Abstract

Atomic force microscopy (AFM) cantilevers are essential components that function both as force sensors and nanoscale interaction tools that play a critical role in AFM capabilities, sensitivity, and precision. Conventional fabrication techniques for probes, that rely on silicon or silicon nitride bulk micro‐machining, generally requires complex fabrication processes associated to low throughput and limited geometric flexibility. Here the development of innovative AFM cantilevers made of silica glass through a novel approach based on selective laser etching is explored, which offers cantilever and tip design flexibility, condense the process into three steps, and reduces the fabrication time and cost while minimizing reliance on complex equipment and clean room facilities. The fabrication and characterization of functional glass cantilevers with thicknesses ranging from 1 to 50 µm and spring constants spanning from 0.02 to 80 N m^−1^ is demonstrated. The fabricated glass probes show excellent performance in both AFM imaging and force spectroscopy applications. The simple and fast fabrication approach, highlights the potential of selective laser etching to produce innovative versatile silica‐based probes for AFM.

## Graphical abstract

Selective laser etching (SLE) enables the monolithic fabrication of customized, fully 3D silica AFM probes integrating both cantilever and tip with controlled 3D geometries. The process allows the production of either sharp or spherical tips with high design flexibility, reproducibility, and yield. The stiffness can be tuned from 0.02 to 80 N m^−1^, supporting both material and cellular force mapping applications.

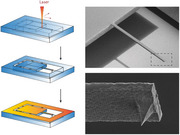

## 1 Introduction

Atomic force microscopy (AFM) is recognized as a very powerful and flexible technique for nanoscale surface imaging and mechanical property measurements. The probe acts as a force sensor and a nanoscale interaction tool playing a critical role in the technique's sensitivity and precision. Therefore, the fabrication of cantilevers and tips with controlled dimensions, mechanical properties and design flexibility, is critical for ensuring high‐performance and measurement accuracy.^[^ ^1^ ^]^

The shape and sharpness of the AFM tip directly influence the lateral resolution, with sharper tips providing clearer, high‐resolution images of surface features, down to the atomic level. Additionally, the mechanical properties, such as spring constant, resonance frequency and quality factor must be precisely controlled. For instance soft cantilevers with low spring constant ( < 1 N m^−1^), allows for the detection of very small forces, making them ideal for studying soft biological specimen, such as cells, membranes, or proteins.^[^ ^2^, ^3^ ^]^ Conversely stiff cantilevers with spring constant (10 – 100 N m^−1^) are preferably used for imaging hard surfaces, as they offer more resistance to surface adhesion and deformation.^[^ ^4^, ^5^ ^]^ Another key mechanical property is the resonant frequency of the cantilever, which determines its dynamic behavior, especially in tapping or non‐contact modes. Cantilevers with high resonance frequencies are less susceptible to thermal noise, so short cantilevers with high resonance frequency can be used in high speed AFM, to study protein and enzymes dynamics.^[^ ^6^, ^7^ ^]^

AFM Cantilevers are conventionally fabricated using silicon, silicon oxide, silicon‐on‐insulator (SOI) or silicon nitride bulk micromachining (MEMS) that have advanced and impacted many field of science and technologies.^[^ ^8^, ^9^ ^]^ These fabrication approaches take an advantage of the batch silicon micromachining techniques developed for integrated circuits (IC) and CMOS. The dimensions of the microcantilevers range from 100 to 500 µm in length and below 5 µm in thickness. These process technologies are typically consisting of series of lithography, material deposition and chemical or physical etching steps, each requires tight control of materials, dimensions, and environmental conditions. The process typically begins with the deposition of a thin film layer—often silicon nitride or silicon—on a substrate. A 2D pattern of the cantilever geometry is then defined on this film using photolithography, which involves coating the surface with a light‐sensitive photoresist, exposing it to ultraviolet light through a patterned mask, and developing the exposed pattern. This step alone requires nanometer‐scale precision and must be aligned with subsequent processing layers. Following photolithography, the exposed areas are etched using Reactive Ion Etching (RIE), KOH etching or Deep Reactive Ion Etching (DRIE). These steps are repeated many times to get the final cantilever. ^[^ ^10^, ^11^, ^12^ ^]^ MEMS technology enables high‐precision and reproducible fabrication of AFM cantilevers, allowing for tight control over their dimensions and mechanical properties. However, this approach is primarily limited to planar, silicon‐based processes, which constrains both the range of usable materials and the complexity of achievable 3D geometries. In particular, fabricating spherical or tapered tips, as well as tips with complex 3D geometry, remains challenging due to the inherent limitations of standard lithographic and anisotropic etching techniques. Recent advances in electrochemical tip formation, for instance through electrodeposition into microfabricated molds, have demonstrated controlled metallic tip growth with nanometer precision.^[^ ^13^ ^]^ These methods yield conductive probes suitable for tribological measurements but are limited in 3D geometric flexibility and often involve complex, multi‐step processing. Alternative approaches for tip fabrication have explored using polymer‐based materials like SU‐8 and PDMS for AFM microcantilevers due to their low stiffness and ease of fabrication.^[^ ^14^, ^15^, ^16^, ^17^, ^18^, ^19^ ^]^ These materials enable high‐throughput production and offer significantly faster imaging in tapping mode—up to ten times faster than silicon‐based cantilevers. However, their tips often lack the sharpness and durability required for contact or resonance modes. Efforts to enhance performances, such as coating SU‐8 tips with graphene, have so far failed to produce sufficiently sharp, wear‐resistant tips, highlighting the need for solutions that combine high‐speed imaging with robust tip geometry. To overcome this, colloidal tips—commonly used for probing the mechanical properties of biological specimens—are often realized by attaching colloidal microbeads to the apex of a tipless cantilever.^[^ ^20^, ^21^, ^22^ ^]^ Similarly, ultra‐sharp or high‐aspect‐ratio tips often require additional post‐processing techniques, such as integration of low‐stress silicon nitride (LSNT) for SU‐8 cantilevers,^[^ ^23^ ^]^ simple field emission induced growth (FEIG),^[^ ^24^ ^]^ SEM or focused ion beam (FIB) milling, which increases both fabrication complexity, reduces throughput and cost.^[^ ^25^, ^26^, ^27^, ^28^, ^29^, ^30^ ^]^

An intriguing approach was proposed by Lee and his co‐workers in 2016,^[^ ^31^ ^]^ to overcome the limitations of silicon based technologies, by involving molding processes for the fabrication of probes with integrated tips consisting in photopolymerizable hydrogels. Such hydrogel‐based cantilevers have widely tunable and low mechanical stiffness suitable for sensitive nanomechanical measurements of soft matter. The process involves using ultraviolet light‐induced curing of a pre‐polymer solution introduced into a mold in order to fabricate the tipless hydrogel cantilever. The tipless microcantilever is then brought into contact with a tip mold filled with a pre‐polymer solution. Curing is achieved by exposing the hydrogel in the tip mold to a secondary ultraviolet light, resulting in a strong bond between the tip and the cantilever, prior to metal coating to enhance reflectivity.

As AFM expands into the characterization of soft materials such as biological tissues and polymers,^[^ ^32^, ^33^ ^]^ the need for probes with lower stiffness and greater design flexibility becomes increasingly evident. Although hydrogel‐based cantilevers offer promising mechanical compliance, their structural and fabrication constraints limit broader applicability. At the same time, commercially available soft silicon‐based probes remain scarce and constrained in both mechanical versatility and geometric customization. This gap highlights the pressing need for multifunctional AFM probes that can span a wide stiffness range and support adaptable tip geometries—ideally enabled by fabrication approaches that are both simplified and inherently versatile.

The recent developments in 3D structuration technologies, in particular 3D printing, has stimulated the manufacturing of microsystems with complex geometries and shapes. In particular, the use of additive manufacturing processes such as high resolution stereolithography or multi‐photon techniques has been used for the fabrication of AFM microcantilevers and tips. N. Alsharif and al.^[^ ^34^ ^]^ have exploited the capabilities of 2 photon polymerization (2PP) for the fabrication of polymer AFM probes at high resolution. The process takes advantage of the flexibility of 2PP to enables the creation of monolithic AFM probes that achieve greater bandwidth in intermittent contact mode, enhancing imaging speed and responsiveness, and to arbitrarily program their modal structure. In a more recent approach, Kramer et al.^[^ ^35^ ^]^ have extended the possibility of fabricating polymer probes by combining stereolithography and 2PP to integrate microfluidic components into the cantilever allowing to combine the functionalities of a standard AFM cantilever along with fluid pipetting.

Additive 3D printing technologies demonstrate their flexibility in controlling 3D design enabling the rapid prototyping and production of small‐ to medium‐scale AFM cantilevers with integrated tips. In particular, 2PP can directly grow polymer tips on pre‐fabricated silicon cantilevers, facilitating on‐chip tip customization and repair.^[^ ^36^ ^]^ Techniques such as high‐resolution stereolithography and two‐photon polymerization (2PP) further allow for precise control over cantilever geometry and the integration of intricate features such as microfluidic channels. However, the performance of these polymer‐based probes is intrinsically limited by the physico‐chemical characteristics of the printable materials. Factors such as a restricted range of available Young's moduli, poor long‐term mechanical stability, sensitivity to ageing, and limited resistance to contact or abrasive forces reduce their suitability for demanding AFM applications. Additionally, the chemical functionalization of such materials remains challenging, thereby constraining their integration into multifunctional or bio‐interactive systems.

In this work, we introduce silica‐based AFM probes fabricated by a new 3D approach, selective laser etching (SLE). SLE is a subtractive 3D micromachining process that involves the modification of a transparent material's internal structure using femtosecond (fs) laser irradiation, followed by selective chemical etching. This technology has been increasingly applied in various fields, such as microfluidics,^[^ ^37^, ^38^, ^39^ ^]^ micromechanics,^[^ ^40^, ^41^, ^42^ ^]^ micropotics,^[^ ^43^ ^]^ and microphotonics.^[^ ^44^ ^]^ Glass the base material in our approach offers several key advantages, including ease of chemical functionalization, optical transparency and biocompatibility which can be a compatible material for AFM cantilevers.

Our objective is to show that SLE is capable of providing multifunctional probes, by producing cantilevers with high aspect ratios, low stiffnesses, and integrated tips with customizable 3D geometries, such as pyramidal and spherical configurations. These features make silica‐based cantilevers versatile for the studies of both soft and stiff specimens. Furthermore, our approach aims to optimize the manufacturing process, by condensate the steps, reduces fabrication time and costs while minimizing dependence on cleanroom environments and complex fabrication equipment, thereby demonstrating the suitability of SLE fabrication approach for AFM probes.

## 2 Results and Discussion

### 2.1 Fabrication Process

The fabrication of the cantilever structures was carried out using Selective Laser Etching (SLE), SLE is a subtractive laser micromachining technique that enables the production of complex 3D architectures in transparent dielectrics such as fused silica.

Laser irradiation initiates nonlinear absorption, leading to permanent structural modifications known as Type II modification (nanogratings), within the irradiated volume of the transparent medium which act as etch‐rate enhancers during subsequent chemical processing.^[^ ^45^, ^46^, ^47^ ^]^

Following laser irradiation, the exposed sample is immersed in an etchant solution such as hydrofluoric acid (HF) or potassium hydroxide (KOH), which selectively dissolves the laser‐modified regions at rates up to thousand times faster than the pristine substrate, allowing selective removal of the defined volumes while preserving the surrounding material.^[^ ^48^ ^]^

**Figure 1:**
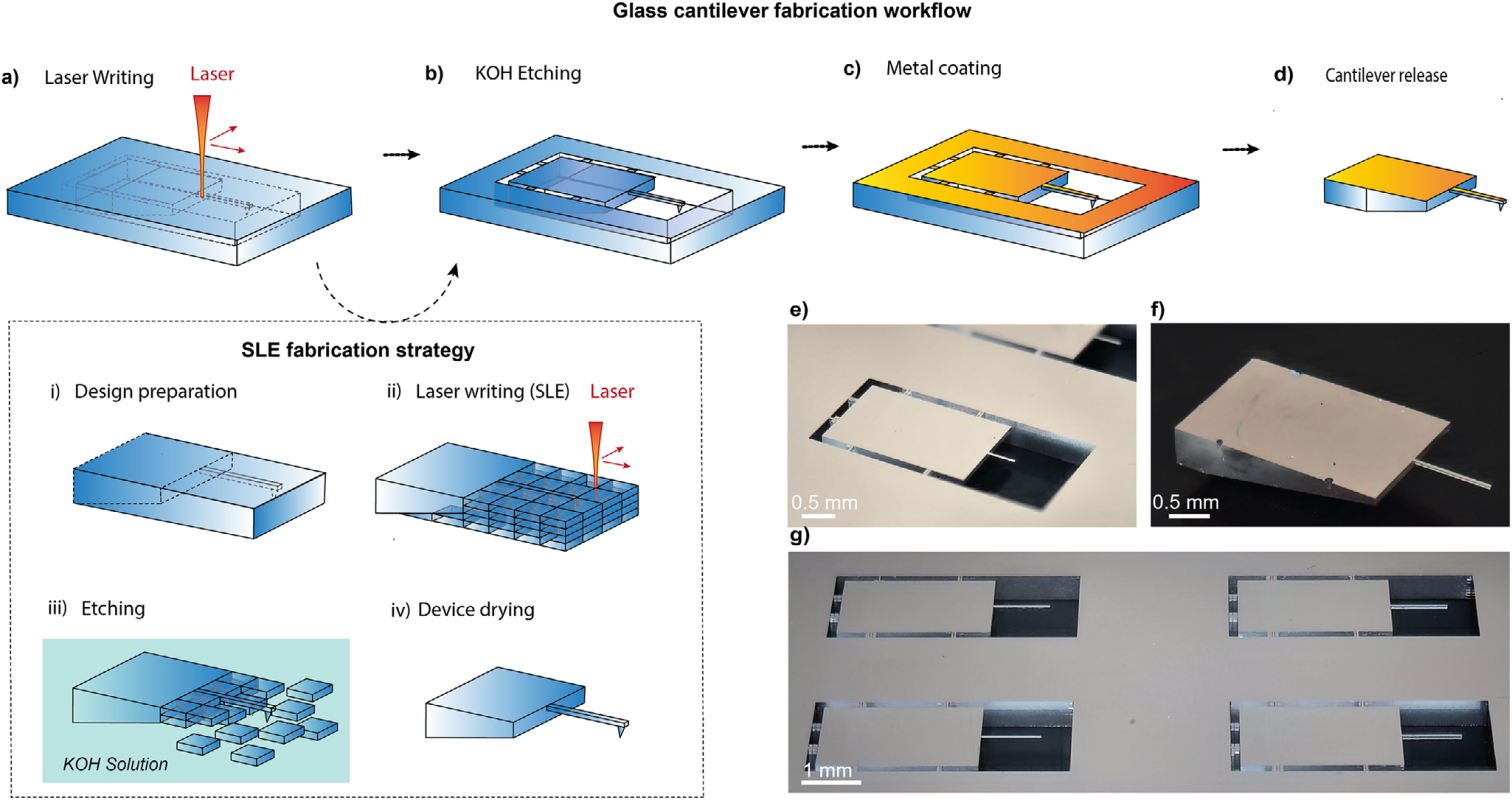
Schematic overview of the cantilever fabrication workflow. a) Illustration of the laser writing strategy bottom‐to‐top irradiation showing a layer inside the glass substrate. i–iv) Overview of the fabrication strategy i) 3D design preparation ii) laser writing strategy highlight the 100 µm × 100 µm square. iii) Illustration of the sample during KOH etching showing sequential detachment of square irradiated blocks iv) sample design after KOH etching and air drying highlight the cantilever and tip b) Illustration of the sample after KOH etching. c) Metallized sample prepared for detachment. d) Final detached cantilever design ready for AFM integration. e) Optical image of the cantilever after metallization. f) Optical image of a detached cantilever ready for AFM integration. g) Optical image showing four cantilevers from the same batch.

In this study, the process began with the computer‐aided design (CAD) of the cantilever geometry, which was then converted into optimized laser scanning trajectories for precise layer‐by‐layer writing inside the fused silica substrate. Laser writing was performed with a bottom‐up irradiation strategy to ensure uniform exposure and reduce debris redeposition. The tip structure was designed directly in the 3D CAD model and fabricated monolithically with the cantilever during the same SLE process. Depending on the targeted application, either pyramidal or spherical geometries were included in the CAD design, positioned at the free end of the cantilever. During laser exposure, the voxel density was increased and the vertical step size reduced to 1 µm in the tip region to ensure continuous material modification and accurate apex shaping. After irradiation, the cantilever structure was released by controlled wet chemical etching in KOH solution. The cantilever was then metallized with a thin bilayer of titanium and gold (Ti/Au, 10/30 nm) to create a reflective backside necessary for laser deflection detection in the AFM optical system. Finally, the fabricated cantilever was integrated into the AFM by means of a specially developed oblique mounting configuration, designed to prevent interference between the probe holder and the sample surface during operation, as illustrated in **Figure** **1**.

Figure 1a–d illustrates the typical design of the cantilevers investigated in this study. We considered cantilevers with simple geometries consisting in rectangular shaped beams. The dotted lines represent the programmed laser scanning trajectories used to inscribe the 2D pattern within the transparent fused silica substrate. The surrounding light blue regions correspond to unmodified material, which remains unaffected by laser exposure and thus defines the precise boundaries of the final cantilever geometry. Six support structures were strategically incorporated to stabilize the cantilever during the etching process, preventing premature detachment. Figure 1(i–iv) shows a detailed view of the fabrication process strategy. For optimization and saving writing time, the etchable volume was discretized into individual 100 µm × 100 µm square blocks, enabling efficient, uniform scanning and minimizing trajectory overlap during layer‐by‐layer fabrication (Figure 1(i,ii)). This segmentation simplified the laser trajectory and allows the writing of a batch of 10 cantilevers in 2 h 37 min that mean ≈16 min per cantilever. Structuring the etchable area into discrete blocks also facilitated more homogeneous and controlled KOH wet etching, as depicted in Figure 1(iii). The KOH etching process was completed in 5 h, and can be applied simultaneously to multiple batches, thereby enhancing throughput and scalability. The cantilevers were then dried in the open air and demonstrated sufficient mechanical resistance to withstand this process (Figure 1(iv)). This contrasts with conventional silicon MEMS cantilevers, which are extremely fragile and typically require supercritical drying using carbon dioxide (CO_2_) to prevent damage caused by capillary forces during liquid evaporation.,^[^ ^49^, ^50^ ^]^ The cantilever design following etching is shown in Figure 1b. Metallization was conducted using a bilayer coating of titanium and gold (Ti/Au, 10/30 nm) to create a reflective backside essential for laser deflection detection in the AFM optical readout system (Figure 1c). In order to integrate the fused silica cantilever into the AFM system, an oblique configuration was specially developed. This configuration prevents the probe holder from touching the surface in front of the cantilever during AFM measurement. Cantilevers were fabricated in parallel on the same substrates. Optical micrographs of the cantilevers before and after detachment from the supporting structures are given in Figure 1e,f,g respectively. This design is the final design of the cantilever with the actual dimensions and configuration. Figure 1d(iv),f illustrates the final cantilever geometry, including its mounting orientation, reflecting the fully optimized design ready for functional AFM integration.

### 2.2 3D Capabilities to Produce Cantilever

This study explored the flexibility of SLE technology to fabricate cantilevers with tunable dimensions and tip geometries to suit a range of applications in materials science and biological force spectroscopy.

As a starting point, a rectangular cantilever was simulated using the known Young's modulus of fused silica (of 70 GPa) to determine the dimensional parameters required to achieve specific spring constants suitable for manipulating biological samples and soft materials (see Figure S1, Supporting Information for detailed calculations).

Simulations targeted a range of cantilever spring constants from 0.75 to 48 N m^−1^ spanning values appropriate for manipulating both biological and material specimens. To achieve this, various cantilever geometries were simulated, using a width from 40 to 100 µm, lengths from 50 µm and 1 mm, and thicknesses ranging from 1 to 20 µm. Based on these simulation specific cantilever dimensions of 40 µm width, 500 µm ‐ 1 mm length, and thicknesses starting at 5 µm, were selected as an initial estimate to be used in the fabrication.

For instance, in the initial fabrication attempt, a cantilever with a target length of 500 µm, width of 40 µm, and thickness of 10 µm yielded a fabricated structure with measured dimensions of 495 µm (L), 30 µm (W), and 5 µm (H). This outcome indicates an over‐etching rate of ≈1 per 100 µm of substrate etched (i.e., 1 µm per hour of etching). Accordingly, a dimensional compensation strategy was implemented to compensate for the 5 h of etching, requiring adjustments of +5 µm for the length and thickness and +10 µm for the width to align fabricated structures with target specifications.

To quantitatively evaluate the fabrication accuracy and reproducibility, we performed a statistical analysis on multiple cantilevers fabricated with different target geometries. The study covered thickness values from 5 to 60 µm, a nominal width of 40 µm, and two nominal lengths (500 and 1000 µm). For each geometry, several cantilevers (*n* = 3) were measured within the same batch, and the process was repeated across three independent fabrication batches (*N* = 3). Two sources of variability were characterized. First, intra‐batch variability was assessed by computing the coefficient of variation (CV) within a single batch, based on repeated cantilevers nominally designed with the same geometry. This metric reflects local process stability for a given fabrication run. Second, inter‐batch variability was quantified by comparing the mean values obtained from the three batches, yielding an inter‐batch (CV†) that captures systematic drift between fabrication runs. These results are summarized in **Table** **1**.

**Table 1:** Summary of dimensional accuracy and variability across fabricated cantilevers. For each target dimension (target value), the table report the programmed design value and the measured mean value. The “CV (%) *” column corresponds to the maximum intra‐batch coefficient of variation calculated from 3 repeated cantilevers within the same batch. The “CV† (%) **” column corresponds to the inter‐batch coefficient of variation, computed form the mean values of three independent batches, and reflects batch‐to‐batch drift in the achieved dimension.

Dimension	Target Value	Programmed value [µm]	Mean Value [µm]	CV [%] *	CV† [%] **
Length (L)	1000 500	1005 505	999,1 500,7	0.15 0.3	0,1 0,2
Width (W)	40	50	40	0.35	0,1
Height (H)	5	10	5,7	20	20
	10	15	10,3	10	10
	15	20	15,9	9	5
	20	25	19,3	7	5
	25	30	24.9	5	5
	35	40	35,2	2	4
	50	55	50,2	2	4
	60	65	59,8	2	2

Overall, the width varies by less than 0.3 % within a given batch, with inter‐batch CV† values on the order of 0.2%, and average widths remaining very close to the target 40 µm. The lengths (500 and 1000 µm nominal) also show high reproducibility, with an inter‐batch CV† well below 1%, and average deviation from the target length of less than 1 µm. As expected, the height dimension is more sensitive to process variations. Over the range studied (5 to 60 µm), the intra‐batch CV and inter‐batch CV vary between less than 2% and 20%, depending on the target height. Despite this higher relative variability, the mean measured height for each nominal value typically remains within ≈1 µm of the intended target.

**Figure 2:**
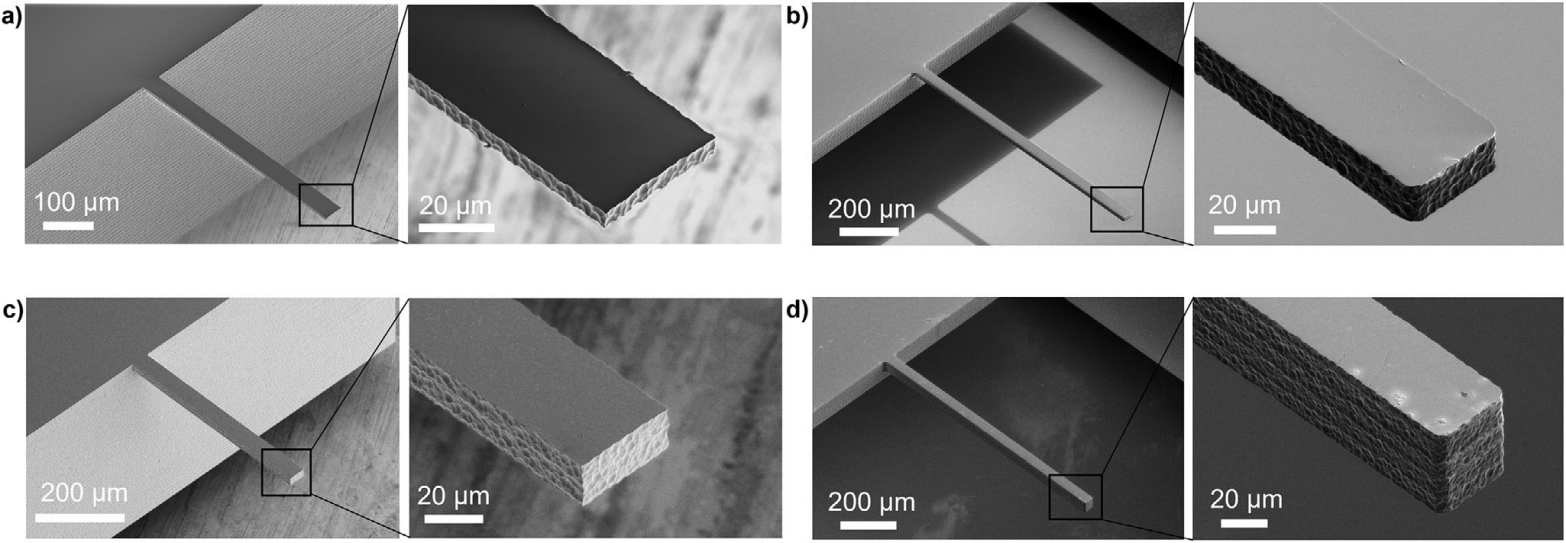
a–d) SEM images of four fabricated cantilevers with varying dimensions, each shown with two views: (left) a larger overview image and (right) a zoomed‐in at the free end of the cantilever. a) cantilever with dimension (L = 500 µm, W = 40 µm, H = 5.7 µm), b) cantilever with dimension (L = 1000 µm, W = 40 µm, H = 15.5 µm), c) cantilever with dimension (L = 1000 µm, W = 40 µm, H = 50 µm), d) cantilever with dimension (L = 500 µm, W = 40 µm, H = 22 µm).

**Figure** **2**a–d display SEM images of four representative cantilevers with widths of 40 µm, lengths of 500 and 1000 µm and four different heights (5.7, 15.5, 22, and 50 µm). These cantilevers have shown excellent mechanical stability, with no visible deformation, and have been successfully integrated into an AFM system and used for AFM measurements. We could demonstrate the fabrication of a cantilever with a low thickness of 1.3 µm (SEM available in Figure S2, Supporting Information) was integrated, and mechanically characterized, displaying stable behavior without observable deformation. The edges of the cantilever shown in Figure 2c display the layered structure resulting from laser writing along the Z‐direction. The SEM images in Figure 2c reveal the layered microstructure along the Z‐direction, a characteristic of the layer‐by‐layer laser writing process, and an edge roughness of 300–500 nm on all cantilevers, while the top surfaces remained smooth as they were unexposed during fabrication.

### 2.3 Laser Reflectivity and Surfaces Roughness

**Figure 3:**
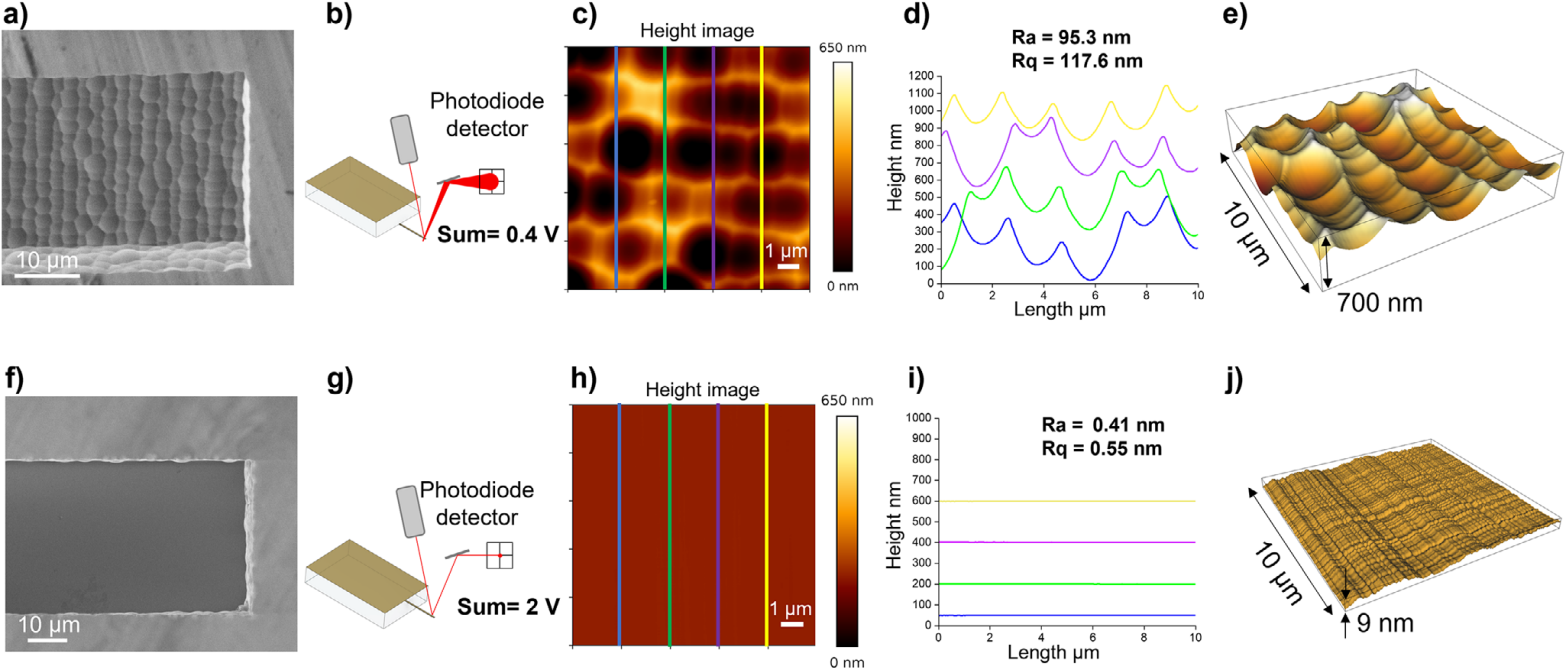
Surface roughness characterization and laser reflectivity analysis of SLE‐fabricated silica cantilevers. a) SEM image showing the metallized rough surface of the cantilever. b) Schematic representation of laser beam reflection from the rough surface to the quadrant photodiodes. c) AFM height image (10 × 10 µm^2^) of the rough metallized surface acquired in contact mode. d) Topographical profiles extracted along the four colored lines indicated in c), illustrating surface valleys resulting from laser exposure. e) 3D projection of the AFM height image shown in (c), highlighting surface roughness. f) SEM image of the opposite (unexposed) side of the cantilever, featuring the smooth metallized surface. g) Schematic showing laser reflection from the smooth surface to the photodiodes, resulting in enhanced optical signal. h) AFM height image (10 × 10 µm^2^) of the smooth surface, showing good surface homogeneity. i) Topographical profiles along the four colored lines indicated in (h), demonstrating minimal roughness. j) 3D projection of the AFM height image shown in (h), confirming the smoothness of the unexposed surface. Contact mode imaging parameters: setpoint – 5 nN, line rate – 0.8 Hz, resolution – 256 × 256 pixels.

An essential component in the operation of an Atomic Force Microscopy (AFM) is the optical detection system which measures cantilever deflection by focusing a laser beam onto the cantilever's backside and detecting its reflection on a position‐sensitive photodiode, typically a quadrant detector (**Figure** **3**b,c).

Initially, the laser reflectivity of the cantilever surface exposed during laser writing and etching was assessed (Figure 3a). After metallization with a Ti/Au bilayer (10/30 nm), laser reflection tests revealed a low sum signal of 0.4 V on the photodiode with significant scattering, preventing stable AFM approach (Figure 3b).

At this stage, the roughness of the metallized cantilever surface was characterized using AFM in contact mode. 2D and 3D height images were obtained to analyze the surface morphology and roughness. The height image presented in Figure 3c quantitatively maps the surface topography, derived from the Z‐piezo displacement necessary to maintain a constant tip–sample interaction force during contact mode AFM. This image reflects the actual surface height variations, where darker regions indicate lower elevations—corresponding to surface valleys. These valleys, distributed across the entire scanned area, contribute significantly to the overall surface roughness and are indicative of regions impacted by laser exposure during fabrication.

Several roughness parameters were extracted, including the arithmetic average roughness (Ra) and the root mean square roughness (Rq), which quantify the average and standard deviation of the surface heights, respectively. The obtained values of Ra = 95 nm and Rq = 118 nm indicate substantial surface roughness, accounting for the light scattering observed on the AFM photodiode. The height‐length graph presented in Figure 3d, extracted from the height image representing the profile of the four colored lines. This graph illustrates the depth of the valleys and how they are presented on the cantilever surface. The distance between peaks is ≈2.1 µm, that matches the programmed distance between the subsequent laser trajectories during laser writing.

The 3D projection of the AFM height image provides additional qualitative information about the surface roughness (Figure 3e). This 3D representation reveals pronounced topographical variations, including elevated and recessed regions throughout the scanned area, highlighting the valleys observed in the height image and confirming the significant roughness of the surface. Based on these characterization results, this cantilever surface is unsuitable for use as a backside reflective surface.

Another roughness characterization was performed on the unexposed surface of the cantilever (Figure 3f). A laser reflection test was conducted to validate this cantilever surface (Figure 1g). The total laser reflection measured on the photodiode was associated to 2 V of laser signal voltage, enabling successful approach and calibration of the AFM system. This test supports reliability of the AFM roughness measurements, which revealed a highly smooth surface with an apparent Ra and Rq < 1 nm, demonstrating excellent homogeneity (Figure 3h–j).

These findings demonstrated that the smooth, unexposed surface of the cantilever—unaffected by laser‐induced roughness—was significantly more effective as a reflective backside for AFM laser detection. To leverage this smoother surface, the cantilever orientation was reversed during integration, which required redesigning the cantilever configuration to avoid physical interference between the holder and the sample surface. This optimized assembly is illustrated in the modified cantilever holder shown in Figure 1d,(iv),f.

### 2.4 Mechanical Characteristics

The mechanical properties of AFM cantilevers are essential for achieving the sensitivity, resolution and precision of AFM measurements, when applied to soft biological samples as well as for hard material specimens.

**Figure 4:**
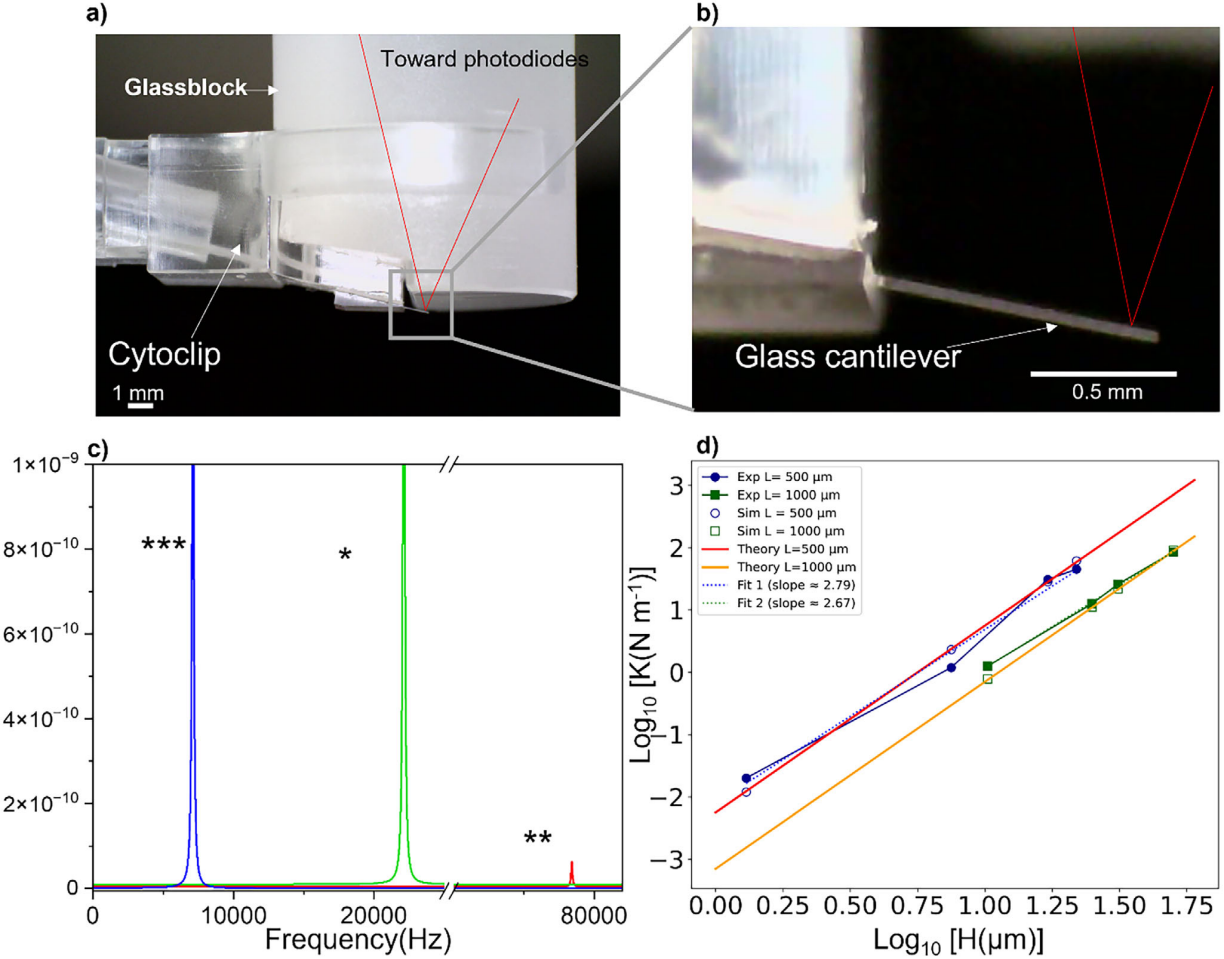
a) Optical image showing the silica AFM cantilever bonded to the Cytoclip holder and integrated into the AFM system. b) A zoom‐in view of the silica cantilever illustrating the laser beam reflecting off the cantilever surface and directed toward the photodiode for deflection detection. c) Thermal noise spectra of three representative AFM cantilevers with different mechanical properties. The spectra were recorded using the NanoWizard 4 system and display the power spectral density (PSD) as a function of frequency. d) Log–log plot of the spring constant as a function of cantilever height for the eight tipless cantilevers listed in Table 2. The blue solid circles and dashed line represent the experimental data and fitted curve, respectively, for cantilevers with a length of 500 µm. Similarly, the green solid square and dashed lines correspond to the experimental data and fit for cantilevers with a length of 1000 µm. The blue and green open markers represent simulation results for cantilevers with lengths of 500 and 1000 µm, respectively. The red and yellow linear curves show the theoretical stiffness curve given by $K=\frac{EwH^{3}}{4L^{3}}$, with w = 40 µm and E = 70 GPa for cantilevers of lengths 500 and 1000 µm, respectively.

Following integration into the AFM system, mechanical parameters of the silica glass cantilever, including spring constant (K), resonance frequency (f_0_) and Quality factor (Q), were evaluated after the integration into the AFM system. **Figure** **4**a,b shown the silica cantilever after being bonded to the cytoclip (FluidFM cantilever holder) and clipped to the AFM glass block, ready for AFM measurements.

**Table** **2** summarizes the measured mechanical parameters obtained using the NanoWizard 4 AFM through thermal noise analysis and sensitivity calibration, with the thermal noise spectra for three representative cantilevers (highlighted in colors in Table 2) shown in Figure 4c.

**Table 2:** Mechanical parameters of the fabricated silica AFM cantilevers, including spring constant (K_exp_), resonance frequency (f_0_), and quality factor (Q), measured using the NanoWizard 4 system via thermal noise analysis and sensitivity calibration. K_sim_ is the spring constant calculated using obtained from finite element models. The cantilevers highlighted in colors correspond to the thermal noise spectra displayed in Figure 4(b): two are tipless, and one (blue) features a pyramidal tip with a square base of 42 µm and a height of 24 µm.

L [µm]	W [µm]	H [µm]	Tip	f0 [kHz]	K exp [N. m^−1^]	K sim [N. m^−1^]	Q	
503.25	40.4	1.3	‐	7.66	0.02	0.012	29	
499	40.2	7.5	‐	22.19	1.18	2.32	145	Figure 4c *
504.6	40.6	17.1	‐	65.82	30.70	28.45	70	
499.1	40.6	21.9	‐	78.39	45.02	61.48	705	Figure 4c **
1000.9	40.5	10.3	‐	11.41	1.26	0.78	78	
1000.9	39.9	24.9	‐	23.92	12.52	11.16	400	
1000.6	39.8	31.2	‐	29.41	25.80	21.72	577	
1002.7	40.2	50.1	‐	44.77	85.65	90.82	843	
1000	42	10	Pyramid	9.356	1	0,77	141	Figure 4c ***

The optical lever sensitivity (in nm. V^−1^) was calibrated using force–distance curves, followed by thermal noise measurements capturing cantilever deflection due to Brownian motion at thermal equilibrium. The resulting deflection data were converted into power spectral density (PSD) spectra using the NanoWizard 4 system, from which resonance frequency and quality factor were extracted using a simple harmonic oscillator fit. The spring constant was determined via the equipartition theorem:

(1) $$\frac{1}{2}k\langle x^{2}\rangle =\frac{1}{2}k_{B}T$$

where *k* is the spring constant of the cantilever, 〈*x* ^2^〉 is the mean square displacement, *k_B_* is Boltzmann's constant, and *T* is the absolute temperature in kelvin.

The fabricated cantilevers exhibited spring constants ranging from 0.02 to 85 N m^−1^, covering a large range of necessary for both biological and material characterization. Although there was a slight difference between the simulation and experimental results, the experimental values remained consistent with the finite element simulations (Table 2), demonstrating strong agreement between the designed and fabricated mechanical properties.

The relationship between the spring constant and the cantilever height was analyzed using log‐log plot, as shown in Figure 4d for 500 and 1000 µm long cantilevers. Both graphs exhibit a linear fit, with slopes of 2.79 and 2.67, respectively, consistent with the theoretical cubic dependence of spring constant on cantilever thickness (*K* ∝ H^3^), as described by the equation:

(2) $$K=\frac{EwH^{3}}{4L^{3}}$$

where E is the elastic modulus, and W, H, and L are the width, height, and length of the cantilever, respectively. This theoretical relationship is illustrated in the graph by the yellow and red linear curves.

The resonance frequency f_0_ of the cantilevers ranged from 7 to 80 kHz in air with quality factor from 28 to 843 providing a narrow bandwidth (≈5% of f_0_), strong energy storage, and high sensitivity, ideal for high‐resolution tapping and non‐contact modes in air.^[^ ^51^, ^52^ ^]^

The stiffer cantilever fabricated was the cantilever with 85.6 N m^−1^ of spring constant exhibiting a resonance frequency of 44.7 kHz and 843 for quality factor. Conversely, the softest cantilever (K = 0.02 N m^−1^), well‐suited for biological applications, exhibited a resonance frequency of 7 kHz with a Q of 28, resulting in a ≈3.7% bandwidth of f_0_, enabling fast response times for real‐time feedback in contact mode while accepting a trade‐off in sensitivity for dynamic imaging.

Overall, the mechanical characterization confirms that the SLE‐fabricated silica cantilevers exhibit the necessary mechanical stability, reproducibility, and performance metrics for reliable AFM measurements across a range of applications, demonstrating the effectiveness of the fabrication strategy in producing high‐quality AFM probes ready for integration and operation.

### 2.5 Pyramidal Tips for Surface Imaging

To validate the silica cantilevers for material characterization, we fabricated cantilevers with integrated pyramidal tips featuring heights from 10 to 30 µm and apex radii down to 33 nm.

**Figure 5:**
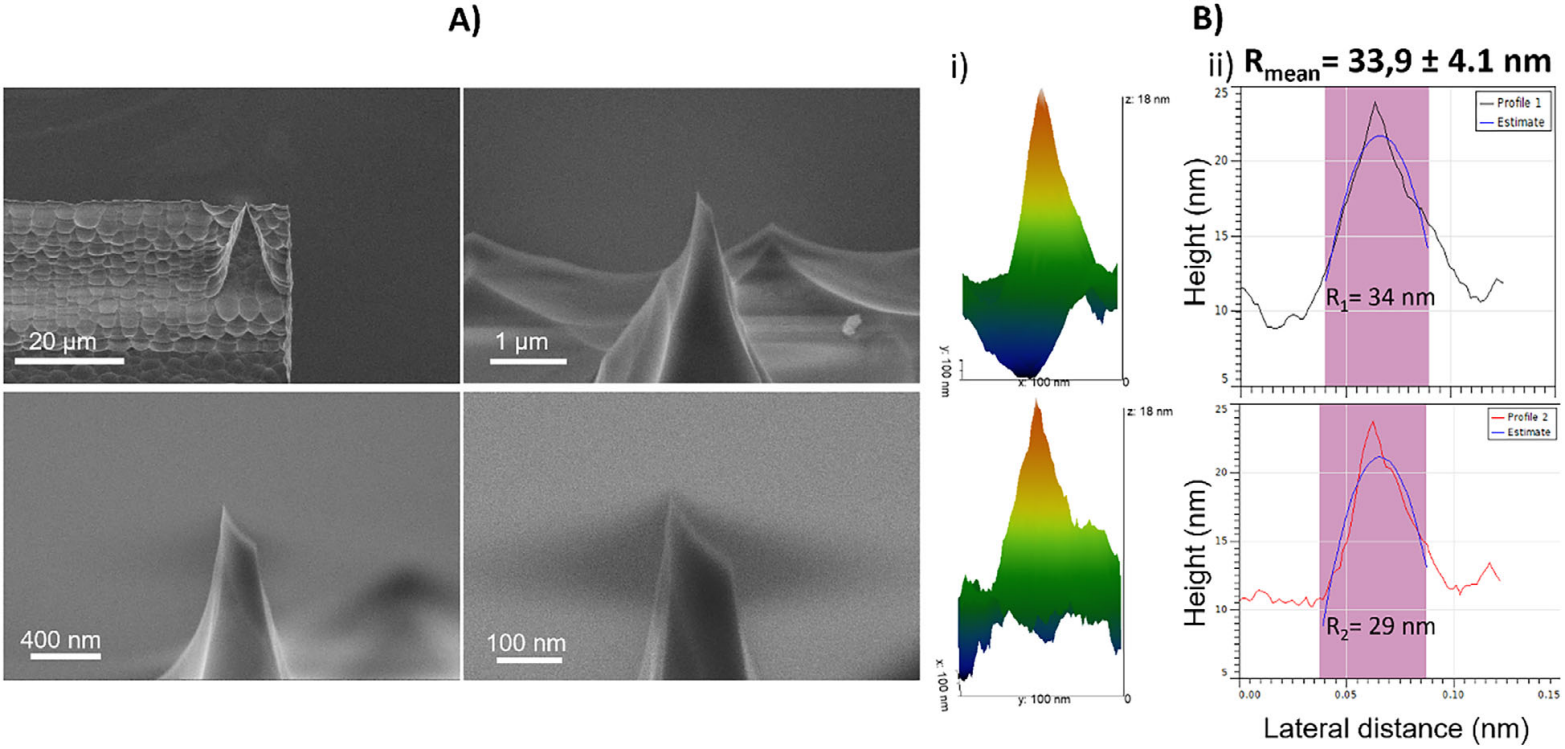
Morphological and geometrical characterization of the AFM tip. A) Four SEM micrographs show the pyramidal tip, and the apex at increasing magnification. B) Tip radius determination in Gwyddion: i) 3D blind‐tip reconstruction (BTR) of the apex obtained in Gwyddion software using a 400 × 400 nm^2^ scan of the Tipcheck calibration grating (Budgetsensor), ii) parabolic fits of two orthogonal apex profiles extracted from the 2D reconstruction. The mean tip radius computed from eight fits is 33.9 ± 4.1 nm. The corresponding AFM cantilever exhibits a length of 1000 µm, width of 40 µm, height of 20 µm, spring constant of 8.27 N m^−1^, and resonance frequency of 19.96 kHz. The pyramid base b = 10 µm and height h = 25 µm.

A quantitative characterization of the curvature radius of the fabricated AFM tips was performed by combining SEM and AFM measurements on a calibration grating. Three cantilevers were analyzed. Prior to AFM measurements, high‐magnification SEM micrographs were acquired at multiple magnifications to verify apex integrity and overall geometry. Each tip was then scanned on a TipCheck calibration grating (BudgetSensors) comprising sharp silicon spikes with nominal apex radii of 5–10 nm. **Figure** **5**A shows four representative SEM micrographs of the pyramidal apex. AFM topographies of the calibration grating were processed in Gwyddion (v2.69) using the Blind Tip Reconstruction algorithm (Villarrubia et al.^[^ ^53^ ^]^), to obtain a 3D reconstruction of the tip. On the reconstructed tip, line profiles through the center of the tip were extracted and fitted with a second‐order polynomial z = ax^2^ +bx+c; the radius of curvature was calculated as R = 1/(2∣a∣). This yielded R = 33.9 ± 4.1 nm, consistent with SEM observations. Characterizations of the two additional tips are provided in Figure S3 (Supporting Information).

**Figure 6:**
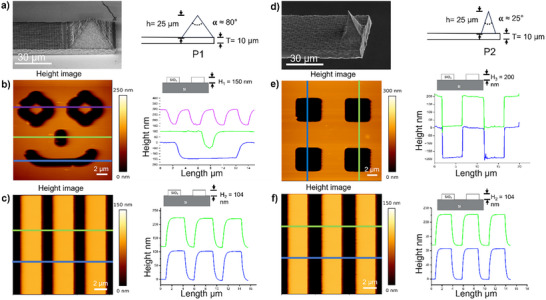
Surface characterization of calibration gratings using custom‐fabricated silica cantilevers. a) SEM of cantilever P1 featuring a pyramidal probe with a wide base (42 µm) and large apex radius (≈243 nm) and a tip angle of ≈80° and spring constant of 0.8 N m^−1^. b) Left: AFM height image obtained using the cantilever P1 by quantitative imaging mode on a trench structure (“smile” with 150 nm depth). Right: Line profiles corresponding to the colored lines in the height image of the “smile” structure. c) Left: AFM height image obtained using the cantilever P1 in contact mode on lines features of 104 nm height with a pitch of 5 µm from the HS 100 MG calibration grating. Right: Line profiles corresponding to the colored lines in the height image. d) SEM of cantilever P2 with a sharper pyramidal tip and smaller apex radius (50 nm) and a tip angle of ≈25° and spring constant of 0.9 N m^−1^. e) Left: AFM height images obtained using the cantilever P2 in contact mode on square features of 200 nm depth with a 10 µm pitch from the P/N 984‐000‐026 calibration grating. Right: Line profiles corresponding to the colored lines in the height image. f) Left: AFM height image obtained using the cantilever P2 in contact mode on lines features of 104 nm height with a pitch of 5 µm from the HS 100 MG calibration grating. Right: Line profiles corresponding to the colored lines in the height image. Contact mode imaging parameters: setpoint – 40 nN, line rate – 0.8 Hz resolution – 512 × 512 pixels. Quantitative imaging mode parameters: setpoint – 50 nN, Z‐length – 0.5 µm, Z‐speed – 50 µm s^−1^, resolution – 128 × 128 pixels.

**Figure** **6**a,d show two representative cantilevers (length: 1000 µm, width: 40 µm, thickness: 10 µm), each equipped with a pyramidal tip of different dimensions and geometries. The performance of these cantilevers was assessed by conducting topographical measurements on various calibration gratings. Cantilever P1 shown in Figure 6a features a pyramidal tip with a base width of 38 µm, a height of 25 µm, and an apex radius of ≈243 nm. A calibration grating HS 100 MG with a pitch of 5 µm and a depth of 104 nm was imaged at a line rate of 0.8 Hz using this cantilever. The resulting profile shows some deviations from linearity, indicating that the broader pyramid base may limit the apex's ability to fully conform to fine surface details (Figure 6c). This limitation was more pronounced on a deeper grating with a 150 nm trench depth (Figure 6b), where profiles showed more pronounced distortions, particularly in the green and violet profiles where the trench width is ≈2 µm. This suggests that the pyramid base interferes with proper contact between the apex and the bottom of the trench, thus affecting imaging fidelity.

In contrast, cantilever P2 (Figure 6d) incorporated a sharper pyramidal tip with a 10 µm square base, 25 µm height, and a ≈50 nm apex radius, comparable to conventional silicon AFM tips. This cantilever was used to image two calibration gratings: one with a 10 µm pitch and 200 nm depth (Figure 6e), and another with a 5 µm pitch and 104 nm step height (Figure 6f), at a 0.8 Hz scan rate yielded profiles with excellent linearity, indicating effective tip engagement with both the top and bottom of the grating structures. Imaging performance was evaluated in comparison with a commercial silicon‐nitride probe (MLCT; nominal tip radius ≈20 nm; spring constant 0.8 N m^−1^), chosen to be close to the spring constant of P2. The full set of comparative topographies and corresponding line profiles is provided in Figure S4 (Supporting Information). These results demonstrate the suitability of sharper pyramidal tips for accurate surface imaging and high‐resolution material characterization, confirming the capability of SLE‐fabricated silica cantilevers to perform reliably in demanding topographical measurements.

### 2.6 Spherical Tips for Cellular Mechanical Characterization

**Figure 7:**
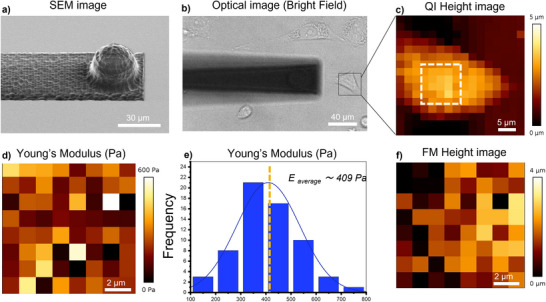
a–f) Images of a PC3‐GFP cell immobilized on a Petri dish acquired using Quantitative imaging and force mapping force spectroscopy. a) Silica rectangular cantilever (8 µm thickness, 40 µm width, 1 mm length) with a spherical tip (23 µm diameter) and a spring constant of 0.3 N m^−1^. b) Optical image showing the cell and the cantilever. c) Height Quantitative imaging image (35 × 35 µm^2^) of the selected cell region indicated by the black square in (b). d) Young's modulus map (10 × 10 µm^2^) of the selected cell region indicated by the white square in (c). e) Quantitative analysis of the cell's young modulus indicating an average value of 409 Pa. f) Height image (10 × 10 µm^2^) acquired using force mapping mode of the selected cell region indicated by the white square in (c). QI parameters: Set point – 5 nN, Z‐length – 7 µm, Z‐speed – 2 µm s^−1^, resolution – 16 × 16 pixels. Force mapping parameters: setpoint – 3 nN, Z‐length – 5 µm, Z‐speed – 10 µm s^−1^, contact time – 0.01 s, scan size – 10 µm, resolution – 8 × 8 pixels.

To demonstrate the potential of the fabricated tips for biological applications, we developed custom silica cantilevers with integrated spherical tips ranging from 10 to 25 µm in diameter. The fabricated cantilever (length: 1000 µm, thickness: 8 µm, width: 40 µm) was designed for compatibility with force spectroscopy and live‐cell imaging (**Figure** **7**a).

To validate their functionality, both force spectroscopy and imaging experiments were conducted on living soft cells (Figure 7b). Cantilever, featuring a 24 µm‐diameter spherical tip, was used to image live PC3‐GFP immobilized cells using quantitative imaging (QI) mode, with a Set point force of 5 nN, Z‐length of 7 µm, Z‐speed of 2 µm s^−1^ and a resolution of 16 × 16 force curves. The resulting image, (Figure 7c), demonstrates a precise topographical mapping of the living cell, confirming the suitability of the spherical probe for non‐destructive biological imaging.

For mechanical characterization, this cantilever was also used to perform force mapping on live PC3‐GFP cells at a Z‐speed of 10 µm s^−1^ and 8 × 8‐pixel resolution (Figure 7f). The setup enabled controlled, precise indentation of cellular structures with a depth of 1.12 µm, allowing measurement of cellular stiffness at 3 nN µm^−1^ (The equation used for the calculation is outlined in Section 4), consistent with values obtained using commercial probes in our recent studies.^[^ ^21^ ^]^ The resulting young's modulus maps confirmed effective stiffness measurement (≈409 Pa) at the single‐cell level (Figure 7d,e). Similar measurements performed using 30 µm diameter spherical tips mounted on 0,2 N m^−1^ cantilever (Figure S5, Supporting Information) confirmed a mean cell stiffness value ≈373 Pa.

These experiments confirm that SLE‐fabricated silica cantilevers with spherical tips are effective for both high‐resolution imaging and biomechanical characterization of living cells, providing reliable, non‐destructive assessment of cellular mechanical properties essential for biological and biomedical research.

### 2.7 Technological Comparison and Scalability Considerations

Conventional AFM probes are typically fabricated from silicon or silicon nitride using microfabrication processes that rely on clean‐room photolithography, oxidation, and anisotropic etching. These methods provide highly reproducible probes with sharp apex radii (often below 30 nm) and low surface roughness, but they are inherently limited to planar, pyramidal geometries and require complex, resource‐intensive infrastructure. In contrast, the selective laser etching (SLE) process developed here enables the monolithic fabrication of fused‐silica cantilevers and tips with full 3D design freedom, using only two compact instruments (a femtosecond laser system and a wet‐etching setup) and without the need for a clean‐room environment. This approach offers a significant simplification of the production workflow and allows the integration of customized geometries or transparent structures that are inaccessible to standard silicon technology.

Although the current prototypes still present slightly higher apex radii and surface roughness compared to commercial silicon tips, these parameters are rapidly improving through process optimization of the laser exposure and etching steps. The present fabrication yield already reaches 90%–100% for ten‐tip batches, demonstrating the robustness of the process. The current estimated throughput is ≈6 × 10 devices per day per SLE system, depending on the programmed geometrical complexity. While the throughput per machine remains moderate, scalability can be achieved by parallelizing production across multiple SLE systems, which is economically more favorable than operating a full clean‐room facility. This architecture provides a realistic pathway toward cost‐effective, distributed, and on‐demand manufacturing of customized AFM probes, highlighting the strong potential of this emerging technology for future commercial applications.

## 3 Conclusion

In this work, we have presented selective laser etching as a novel approach for the fabrication of AFM cantilevers. A three‐step process: i) laser irradiation, ii) KOH etching, and iii) Ti/Au metallization; was employed to produce innovative silica‐based cantilevers. A batch of 10 custom cantilevers can be fabricated in 2 h and 37 min. We believe that this time can be further reduced by a factor of 2 to 4 by optimizing fabrication parameters such as design, laser power, and spot size.

Our method only involves 3 fabrication steps associated to 3 different equipment and results in a fabrication time and energy consumption reduction of ≈50% and a cost reduction ≈25%, compared to conventional cantilevers microfabrication approaches (based on equipment usage and operational data from our clean room). Standard silicon‐based cantilever fabrication involves repeated cycles of photolithography, deposition, and etching steps, often relying on up to 15 different machines, including ovens, plasma tools, and etching systems—and necessitating the use of toxic gases and large volumes of ultrapure water, all of which contribute to significant environmental and economic burdens. In contrast, our approach uses no gases, only a small amount of water, and can be carried out outside of a clean room, offering a cost‐effective and environmentally sustainable alternative for producing high‐performance AFM cantilevers.

Our process allows for precise control of cantilever geometry, yielding thicknesses from 1 to 50 µm and a broad range of spring constants (K = 0.02 to 80 N m^−1^) and resonance frequency from 7 to 78 kHz, suitable for imaging both soft biological samples and rigid materials.

The surface imaging capability of these silica cantilevers was demonstrated in contact mode AFM on different calibration gratings with pyramidal tips. The spherical tip enabled controlled and precise probing of the cellular structures of PC3‐GFP cells with indentation depth of 1.12 µm and measured cell membrane elastic modulus of 409 Pa.

The silica‐based AFM cantilevers offers several key advantages, including optical transparency, ease of chemical functionalization, and biocompatibility. Therefore, the optical properties of silica make these cantilevers compatible with techniques such as scanning near‐field optical microscopy (SNOM).^[^ ^54^ ^]^ Beyond material aspects, the SLE fabrication approach enables the freedom of customized 3D cantilever and tip geometries, including spherical, tapered, cylindrical and other complex 3D structures. These capabilities expand the potential of AFM by enabling the development of customized probes integrating optical or microfluidic components opening pathways for multimodal nanoscale characterization.

## 4 Experimental Section

### Substrate and Chemicals

Fabrication was performed on fused silica glass substrates *(JGS2, Microchem)*, supplied as into square pieces measuring 30 × 30 mm^2^ with a thickness of 500 ± 25 µm. The chemicals used in the process included potassium hydroxide (KOH, 6 mol L^−1^), acetone, and isopropanol (IPA).

### Design Preparation CAD

The silica probe (holder, cantilever, and tip) 3D models were created using SolidWorks 2024 (Dassault Systèmes, France) and exported in STEP format. A set of ten cantilevers was constructed as an array assembled on a glass substrate. Each probe assembly comprised:

i) a rectangular probe holder measuring 2.7 × 1.7 mm^2^ with a thickness of 500 µm, featuring a 10° cut angle on the side where the cantilever is located, designed to ensure the holder does not contact the surface before the cantilever;

ii) a rectangular cantilever defined by dimensions L*, W*, and H*, which are designed to yield the desired final dimensions L, W, and H after fabrication. Specifically, the adjusted dimensions are L* = L + 5 µm, W* = W + 10 µm, and H* = H + 5 µm.

iii) Six supporting structures, each measuring 150 × 40 µm^2^ with a thickness of 70 µm strategically positioned to stabilize and protect the cantilever during the fabrication process, up until final detachment and integration.

iv) A tip, either spherical or pyramidal modeled at the free end of the cantilever.

### Laser Induced Modification Process

Laser exposure of glass substrates was carried out using a Femtika Laser Nanofactory system (Femtika.Ltd, Lithuania), equipped with an ultrafast Yb:KGW femtosecond laser (1030 nm central wavelength, 700  fs pulse duration, 610  kHz repetition rate).

This system enables 3D microfabrication through a high‐precision XYZ translation stage combined with galvanometric scanners for rapid beam steering. The laser beam was focused into the volume of the glass substrate using a 20× microscope objective with a numerical aperture (NA) of 0.45, producing a focal spot ≈3 µm in diameter in the XY plane and ≈14 µm along the Z‐axis.

In the process, the cantilever and the tip were designed and fabricated simultaneously as a single, monolithic component: the full STEP geometry (holder, cantilever, and tip) is written in one laser‐exposure job within the substrate. The laser was operated in a spot‐by‐spot writing mode at a scanning speed of 25 mm s^−1^. A vertical (Z) step size of 7 µm was used for cantilever structuring while a 1 µm step size was used for tip exposure. The hatching distance defined as the lateral spacing between consecutive scan lines was set to be 2 µm.

The writing process was performed in an ambient air condition, on a vibration‐isolated stage to maintain alignment stability. The focal plane was pre‐calibrated, and precise depth control was ensured using integrated Femtika's imaging system.

The Femtika's software provides full 3D visualization of the laser writing process, allowing precise definition and verification of the scanning paths within the fused silica substrate. (Figure S6, Supporting Information)

### Etching and Post Processing

After the laser modification process, the substrate was immersed in 6 mol L^−1^ potassium hydroxide (KOH) solution at 90 °C for 5 h. Following this step, the oven was turned off, and the substrate was allowed to cool down in the oven until it reached 50 °C. The substrate was then removed, cleaned with deionized (DI) water, followed by a resistivity measurement performed to verify the effectiveness of the rinsing process. This ensures that no residual ions (such as K⁺ or OH^−^) remain on the surface. Finally, the substrate was then left to dry in ambient conditions.

### Cantilever Metallization

In order to enable laser reflection on the cantilever, a titanium/gold (Ti/Au) bilayer with thicknesses of 10 and 30 nm, respectively, was deposited. Prior to metallization, a cleaning cycle involving Acetone, DI water, and isopropanol, followed by 2 min oxygen plasma treatment at 200 W to remove any remaining residues and improve metal adhesion were conducted. The metal layers were deposited by thermal evaporation in a vacuum chamber maintained at a pressure of 1 × 10^−6^ mbar, with a deposition rate of 0.2 nm s^−1^, using an EVA 600 system (Alliance Concept).

### Characterization

*SEM*: Scanning electron microscopy (SEM) was performed using a SEM VERIOS 5 UC in secondary electron detection mode. For non‐metallized samples, an acceleration voltage of 1 kV and an intensity of 13 pA were used, whereas for metallized cantilevers, a voltage of 5 kV and an intensity of 50 pA were utilized. Dimensional measurements were conducted using Fiji an open‐source image processing software.

*Characterization of Cantilever Roughness*: Surface roughness characterization was performed using a NanoWizard 4 atomic force microscope (Bruker, Germany) operating in contact mode. Scans were acquired over 10 × 10 µm^2^ areas with a resolution of 256 × 256 pixels at a scan rate of 0.8 Hz. A cantilever equipped of 10 µm height pyramidal tip (FluidFM Nanosyringe‐Cytosurge) and a nominal spring constant of 2.8 N m^−1^ was used for imaging. Topographical data and force curves were processed using JPK analysis software. A first‐order plane fit was applied to each image to correct for sample tilt. Surface morphology was evaluated to calculate the arithmetic mean roughness (Ra) and root mean square roughness (Rq).

### AFM

*Mounting of the Glass Cantilever onto the AFM Head*: To integrate the glass cantilevers into AFM setup, previously Cytosurge's FluidFM used cantilevers were unmounted from their Cytoclip system. A UV‐curable adhesive (Polytec PT GmbH, UV 2108) was then applied to securely bond the glass cantilever to the Cytoclip. Once the adhesive was cured and the connection established, the assembled cantilever was mounted into the AFM using the standard probe holder, making it ready for experimental use.

*Spring Constant Measurement*: Cantilever calibration was performed using a glass slide as a reference surface. The laser was precisely aligned on the metallized apex of the cantilever, which was then approached toward the glass slide at a speed of 7.5 µm s^−1^ until contact was established with a deflection setpoint of 1 V. Sensitivity was determined through the deflection signal, and the spring constant was calculated using thermal noise analysis. The final spring constant value was obtained by averaging the results from three consecutive measurement cycles.

*AFM Imaging*: To validate the imaging capabilities of the cantilevers, standard calibration gratings were scanned, including Digital Instruments gratings (P/N 498‐000‐026) with a 200 nm step height and 10 µm lateral pitch, and HS‐100MG gratings (Budget Sensors) featuring a 104 nm measured step height and 5 µm pitch. Imaging was performed in contact mode at a line rate of 0.8 lines. s^−1^ with a resolution of 512 × 512 pixels, and in quantitative imaging mode at a scan speed of 50 µm s^−1^ with resolution 128 × 128 pixels. The setpoint force for each image is specified in the corresponding figure caption in the results section. Various scan sizes (50, 20, and 10 µm) were used to capture different regions of the gratings. The acquired topography images were analyzed to compare measured step heights and lateral dimensions with the manufacturer's nominal values, to confirm the functionality and imaging accuracy of the glass cantilevers.

*Force Spectroscopy*: To evaluate the effectiveness of spherical tips in probing the cellular structures of individual living cells via force spectroscopy, both force mapping and Quantitative Imaging (QI) modes measurements were performed on immobilized PC3‐GFP cells. Force mapping was performed at a z‐speed of 10 µm s^−1^ with spatial resolutions of 4 × 4 and 8 × 8 pixels over a 10 × 10 µm^2^ scan area. QI measurements were conducted at z‐speeds of 2 and 10 µm s^−1^, with resolutions of 16 × 16 and 32 × 32 pixels across the same scan area. The resulting force‐distance curves and topographical images were analyzed using JPK Data processing software. Mechanical parameters, such as young's modulus, indentation depth, and cell stiffness, were quantified. These parameters were calculated using the Hertz contact model for a spherical indenter, based on the equation $F=\frac{4}{3}\cdot \frac{E_{s}}{1-\nu_{s}^{2}}\cdot \sqrt{R}\cdot \delta^{3/2}$, where F is the applied force, R is the radius of the spherical tip, E_s_ is the young's modulus of the sample (Pa), ν_s_ is the Poisson's ratio of the sample, and δ is the indentation depth. The extracted values were subsequently compared with previously reported data obtained using commercial AFM probes.

### Culture Cell

The PC3‐GFP cell line was cultured in RPMI medium, containing L‐glutamine, HEPES buffer and phenol red (Gibco, Thermo Fisher Scientific Inc.) with 10% fetal bovine serum (FBS, Gibco, Thermo Fisher Scientific Inc.), 1% penicillin‐streptomycin (Gibco, Thermo Fisher Scientific Inc.) and 1% geneticin (G418, Gibco, Thermo Fisher Scientific Inc.). The cell line was grown in an incubator at 37 °C and 5% CO2. The PC3‐GFP cells were then seeded into a 9 cm^2^ TPP tissue culture dish at a concentration of 25 000 cells per cm^2^ and incubated overnight in complete culture medium to allow for cell adhesion and recovery. During AFM experiments, the cells were maintained at physiological conditions, with the sample stage kept at 37 °C and a controlled atmosphere of 5% CO_2_ to ensure cell viability throughout the measurements.

## Conflict of Interest

The authors declare no conflict of interest.

## Supporting information



Supporting Information

## Data Availability

All data are available in the main text or the supplementary materials.
