# Supplementary material for: 3D Customized Silica‐Based AFM Probes Fabricated by Selective Laser Etching
Source: Small Methods. 2026 Jan 19;10(4):e01772. doi: 10.1002/smtd.202501772 (PMC12929932; doi:10.1002/smtd.202501772)
Supplement: Supplementary file 1 — Supporting Information [file SMTD-10-e01772-s001.docx]

Supporting Information


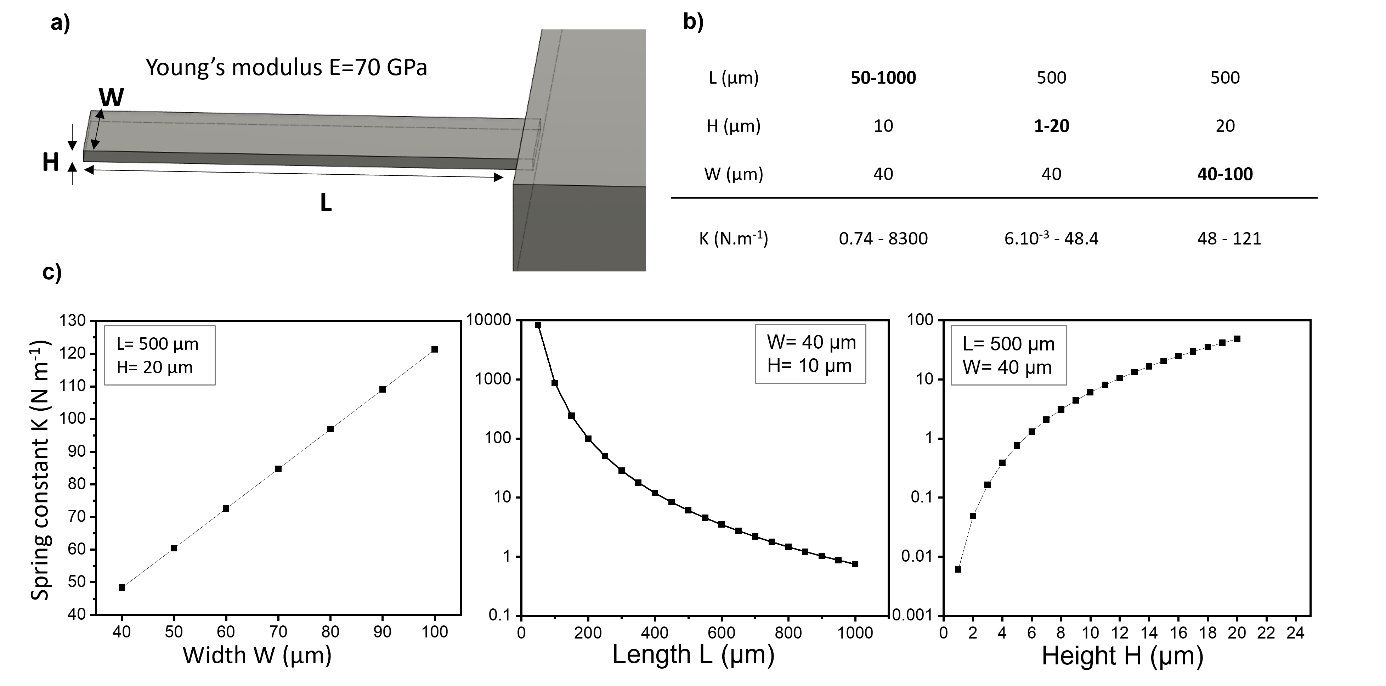


Figure S1. *(a) Represents the rectangular cantilever design simulated with Young’s modulus E = 70 GPa. (b) The table shows the resulting values of the spring constant obtained by fixing two parameters and varying the third. (c) Displays three graphs of the logarithm of the spring constant as a function of the cantilever dimensions.*


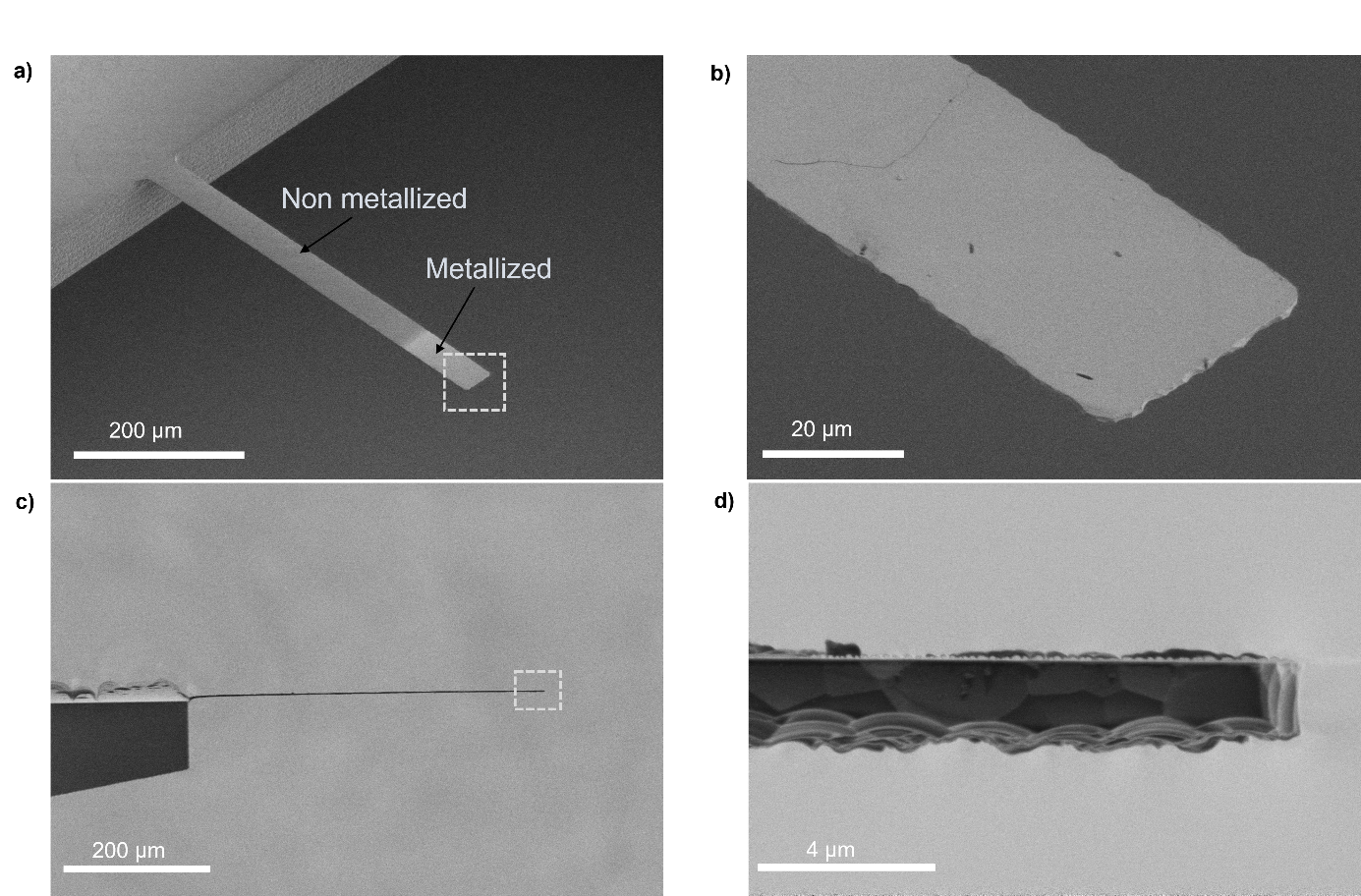


**Figure S2.** *SEM images of a tipless cantilever with dimensions of 500 µm length, 40 µm width, and 1.3 µm height. (a) SEM image showing metallized and non-metallized surfaces. (b) Zoomed-in view of the cantilever end. (c) Side view of the entire cantilever. (d) Zoomed-in side view of the cantilever end.*


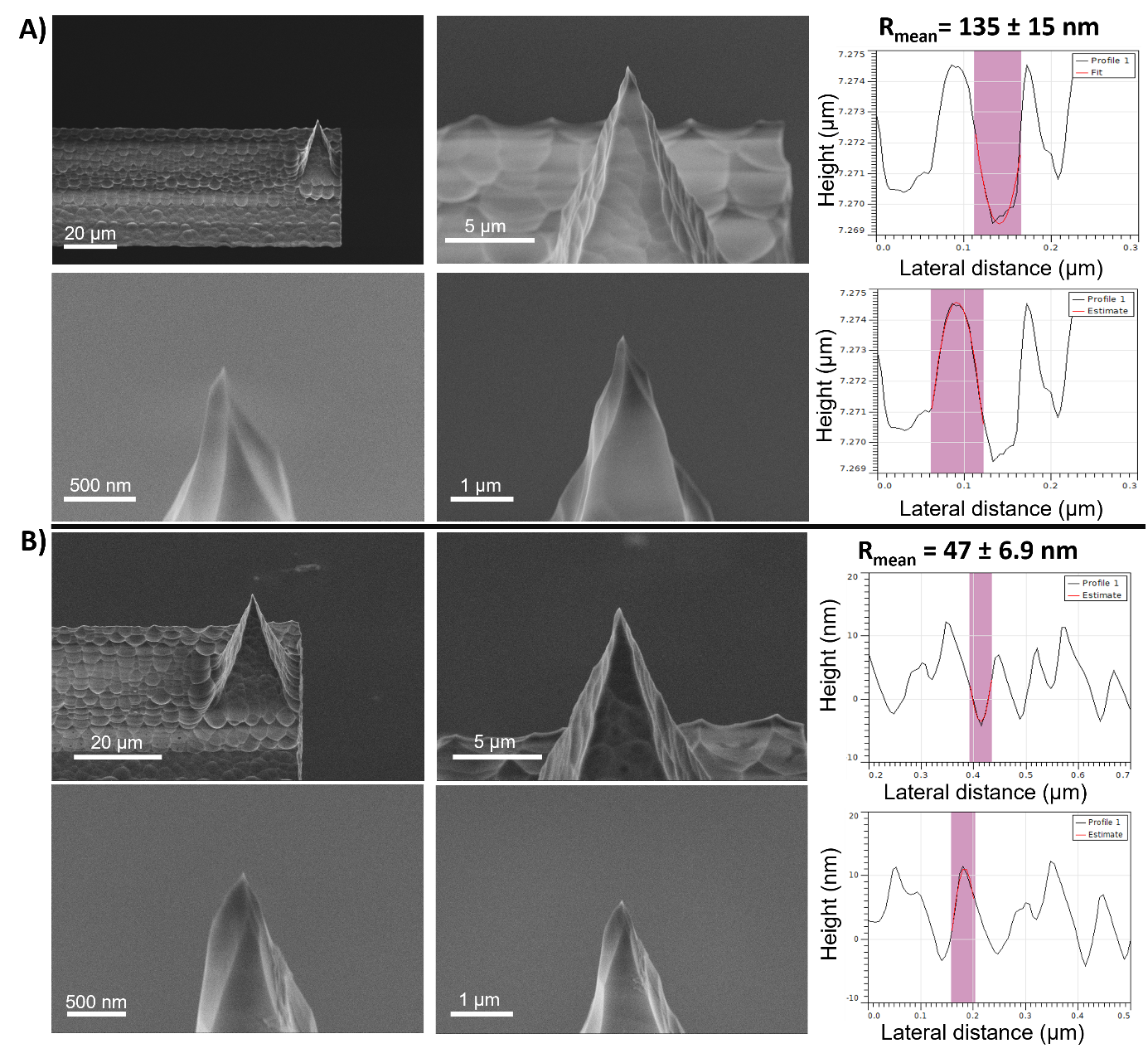


**Figure S3**. Morphological and geometrical characterization of two AFM tips. (A) Left: four SEM micrographs showing the pyramidal tip and apex at increasing magnifications. Right: representative AFM cross-sections of TipCheck spikes used for curvature analysis. The apparent tip radius was determined by fitting a second-order polynomial (z=ax^2^+bx+c) to narrow regions around the apex of five spikes. The radius of curvature was then calculated from the fit coefficient as R=1/(2∣a∣). The resulting mean radius was 135 ± 15 nm (mean ± SEM; n=5). Pyramid base b= 12 µm, height h= 30 µm. (B) Same analysis performed on a second tip, combining SEM imaging and AFM-based cross-sectional fitting. The extracted mean curvature radius was 47± 6.9 nm (mean ± SEM; n=5). Pyramid base b= 15µm, height h= 35 µm.


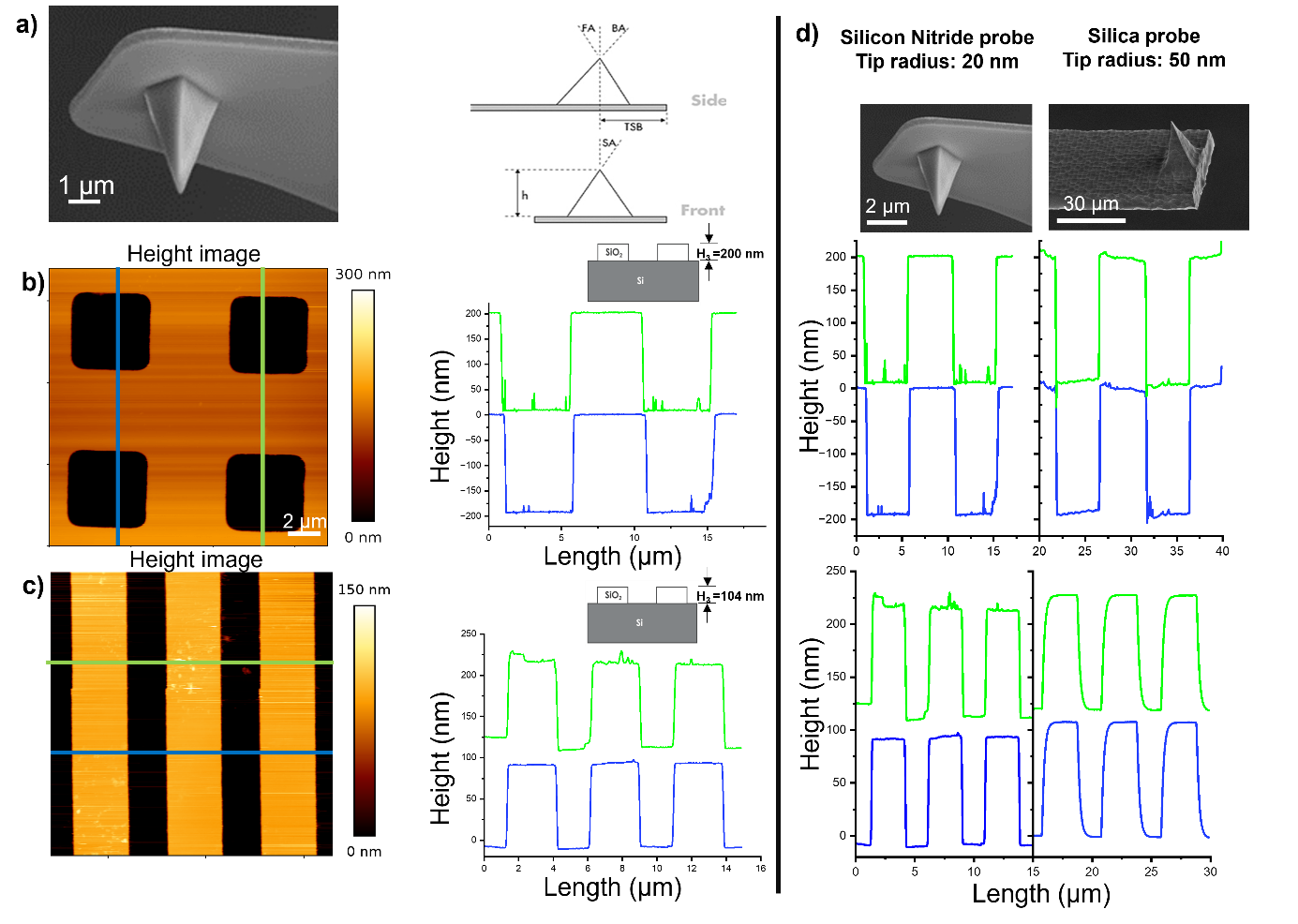


**Figure S4**. Surface characterization of calibration gratings using commercial silicon nitride cantilever (MLCT, Bruker). (a) SEM of the cantilever F of MLCT featuring a pyramidal probe (tip height 2.5–8.0 µm; front angle FA = 15 ± 2.5°; back angle BA = 25 ± 2.5°; side angle SA = 17.5 ± 2.5°; nominal tip radius ≈ 20 nm; adapted from the Bruker website) and a spring constant of. (b) Left: AFM height images obtained in contact mode on square features of 200 nm depth with a 10 µm pitch from the P/N 984-000-026 calibration grating. Right: Line profiles corresponding to the colored lines in the height image. (c) Left: AFM height image obtained in contact mode on lines features of 104 nm height with a pitch of 5 µm from the HS 100 MG calibration grating. Right: Line profiles corresponding to the colored lines in the height image. (d) Comparison of (silica-silicon nitrides) probes imaging performance: plots of the height–length profiles for the commercial MLCT (Silicon nitride probe) and the fabricated silica probe P2. Contact mode imaging parameters: setpoint – 40 nN, line rate – 0.8 Hz resolution – 512 × 512 pixels.


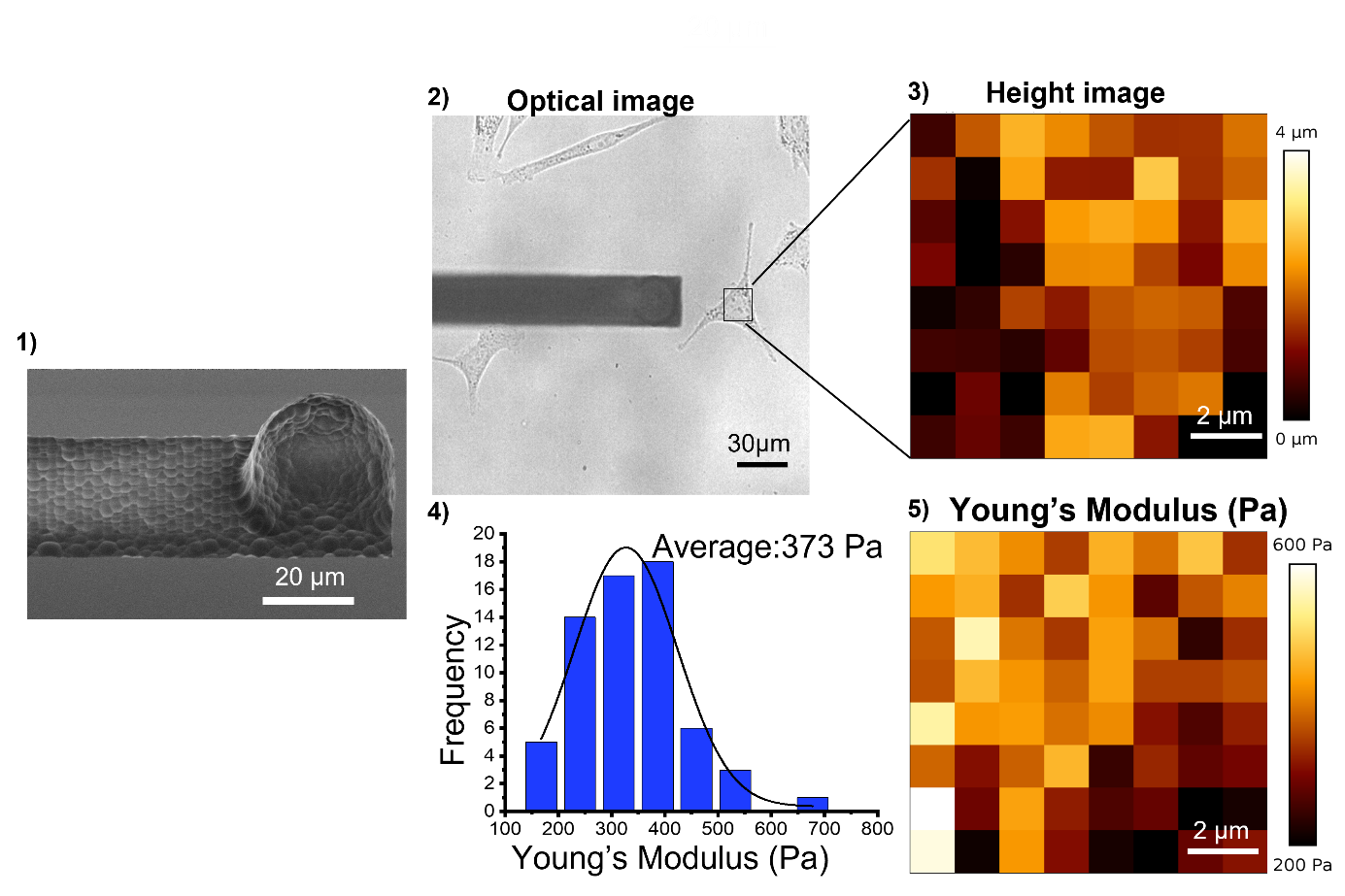


**Figure S5.** *Images of a PC3-GFP cell immobilized on a Petri dish acquired using force mapping mode. (1) Silica rectangular cantilever (8 µm thickness, 30 µm width, 1 mm length) with a spherical tip (30 µm diameter) and a spring constant of 0.2 N.m^-1^. (2) Optical image showing the cell and the cantilever. (3) Height image (10 × 10 µm²) of the selected cell region indicated by the square in (2). (4) Quantitative analysis of the cell's young’s modulus. (B5) Young’s modulus map with an average value of 373 Pa. Force mapping parameters: setpoint – 3 nN, Z-length – 5 µm, Z-speed – 10 µm. s^-1^, contact time – 0.01 s, scan size – 10 µm, resolution – 8 × 8 pixels.*


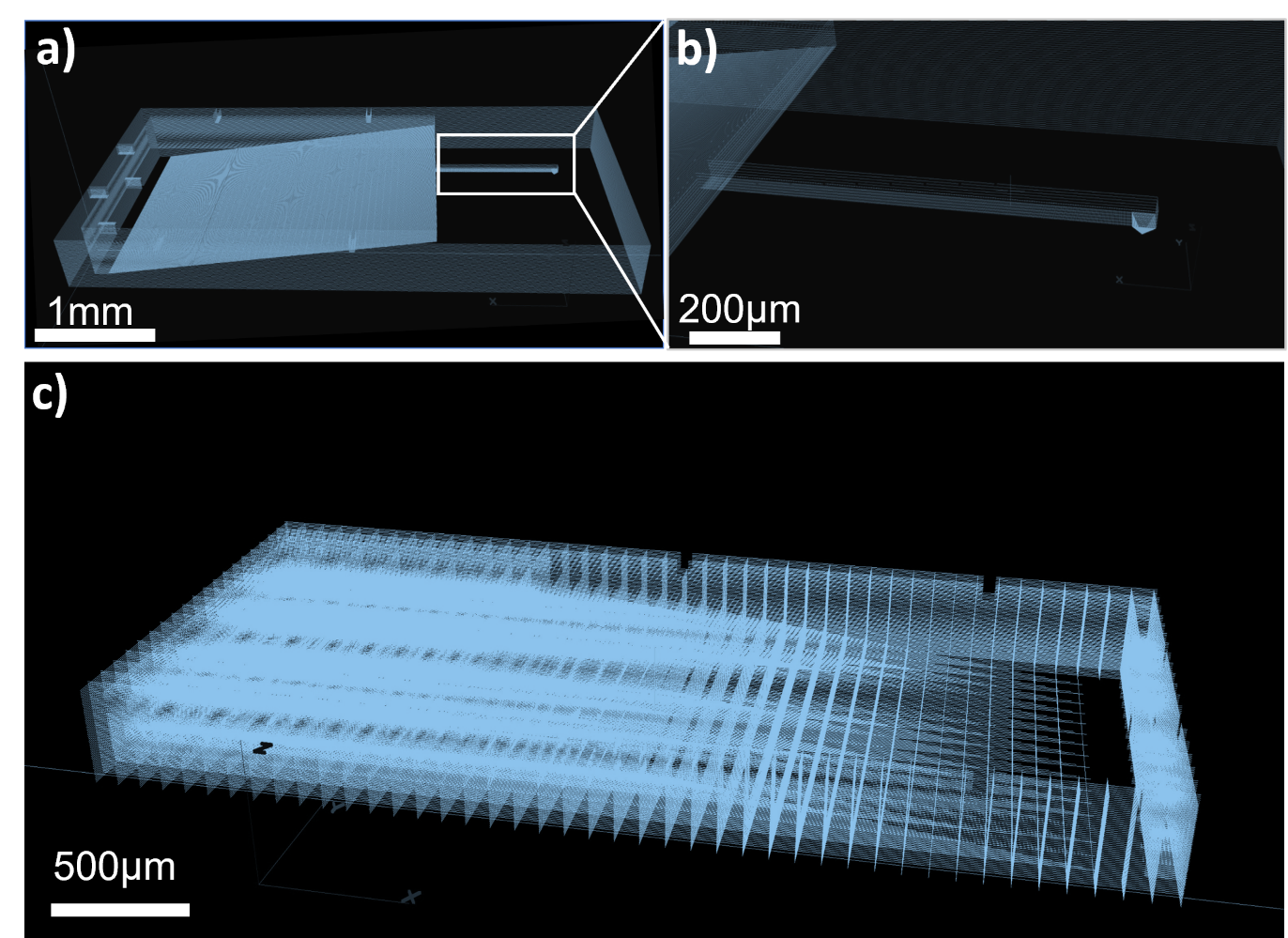


**Figure S6.** Visualization of the 3D laser-writing process in fused silica using the Femtika Laser Nanofactory system. (a) 3D rendering of the complete laser-written volume corresponding to the final AFM cantilever and holder geometry. (b) Zoom view of the cantilever region from (a) showing the cantilever and tip. (c) Visualization of the laser-writing trajectory defining the scanning path used to generate the modified volume prior to selective etching.

[1] J. S. Villarrubia, ‘Scanned probe microscope tip characterization without calibrated tip characterizers’, *J. Vac. Sci. Technol. B Microelectron. Nanometer Struct. Process. Meas. Phenom.*, vol. 14, no. 2, pp. 1518–1521, Mar. 1996, doi: 10.1116/1.589130.

[2] A. Alessandrini and P. Facci, ‘AFM: A versatile tool in biophysics’, *Meas Sci Technol*, vol. 16, pp. 65–92, Jul. 2005, doi: 10.1088/0957-0233/16/6/R01.

[3] M. Krieg *et al.*, ‘Atomic force microscopy-based mechanobiology’, *Nat. Rev. Phys.*, vol. 1, no. 1, pp. 41–57, Nov. 2018, doi: 10.1038/s42254-018-0001-7.

[4] S. Oraby and A. Alaskari, *Atomic Force Microscopy (AFM) Topographical Surface Characterization of Multilayer-Coated and Uncoated Carbide Inserts*, vol. 46. 2011.

[5] S. Gonda *et al.*, ‘Accurate topographic images using a measuring atomic force microscope’, *Appl. Surf. Sci.*, vol. 144–145, pp. 505–509, Apr. 1999, doi: 10.1016/S0169-4332(98)00851-4.

[6] T. Ando, T. Uchihashi, and N. Kodera, ‘High-Speed AFM and Applications to Biomolecular Systems’, *Annu. Rev. Biophys.*, vol. 42, no. 1, pp. 393–414, May 2013, doi: 10.1146/annurev-biophys-083012-130324.

[7] N. Kodera, T. Kinoshita, T. Ito, and T. Ando, ‘High-Resolution Imaging of Myosin Motor in Action by a High-Speed Atomic Force Microscope’, in *Molecular and Cellular Aspects of Muscle Contraction*, vol. 538, H. Sugi, Ed., in Advances in Experimental Medicine and Biology, vol. 538. , Boston, MA: Springer US, 2003, pp. 119–127. doi: 10.1007/978-1-4419-9029-7_11.

[8] A. S. Algamili *et al.*, ‘A Review of Actuation and Sensing Mechanisms in MEMS-Based Sensor Devices’, *Nanoscale Res. Lett.*, vol. 16, no. 1, p. 16, Jan. 2021, doi: 10.1186/s11671-021-03481-7.

[9] B. O. Alunda and Y. J. Lee, ‘Review: Cantilever-Based Sensors for High Speed Atomic Force Microscopy’, *Sensors*, vol. 20, no. 17, Art. no. 17, Jan. 2020, doi: 10.3390/s20174784.

[10] Q. Yu, G. Qin, C. Darne, C. Cai, W. Wosik, and S.-S. Pei, ‘Fabrication of short and thin silicon cantilevers for AFM with SOI wafers’, *Sens. Actuators Phys.*, vol. 126, no. 2, pp. 369–374, Feb. 2006, doi: 10.1016/j.sna.2005.10.019.

[11] D. Saya, K. Fukushima, H. Toshiyoshi, G. Hashiguchi, H. Fujita, and H. Kawakatsu, ‘Fabrication of single-crystal Si cantilever array’, *Sens. Actuators Phys.*, vol. 95, no. 2, pp. 281–287, Jan. 2002, doi: 10.1016/S0924-4247(01)00742-7.

[12] S. Kuwahara, S. Akita, M. Shirakihara, T. Sugai, Y. Nakayama, and H. Shinohara, ‘Fabrication and characterization of high-resolution AFM tips with high-quality double-wall carbon nanotubes’, *Chem. Phys. Lett.*, vol. 429, no. 4, pp. 581–585, Oct. 2006, doi: 10.1016/j.cplett.2006.08.045.

[13] M. Milczarek, D. M. Jarząbek, P. Jenczyk, K. Bochenek, and M. Filipiak, ‘Novel paradigm in AFM probe fabrication: Broadened range of stiffness, materials, and tip shapes’, *Tribol. Int.*, vol. 180, p. 108308, Feb. 2023, doi: 10.1016/j.triboint.2023.108308.

[14] V. Seena, A. Fernandes, P. Pant, S. Mukherji, and V. Ramgopal Rao, ‘Polymer nanocomposite nanomechanical cantilever sensors: material characterization, device development and application in explosive vapour detection’, *Nanotechnology*, vol. 22, no. 29, p. 295501, Jun. 2011, doi: 10.1088/0957-4484/22/29/295501.

[15] A. Johansson, M. Calleja, P. A. Rasmussen, and A. Boisen, ‘SU-8 cantilever sensor system with integrated readout’, *Sens. Actuators Phys.*, vol. 123–124, pp. 111–115, Sep. 2005, doi: 10.1016/j.sna.2005.03.025.

[16] V. Seena, A. Nigam, P. Pant, S. Mukherji, and V. R. Rao, ‘“Organic CantiFET”: A Nanomechanical Polymer Cantilever Sensor With Integrated OFET’, *J. Microelectromechanical Syst.*, vol. 21, no. 2, pp. 294–301, Apr. 2012, doi: 10.1109/JMEMS.2011.2175703.

[17] D.-S. Kim, Y.-J. Jeong, B.-K. Lee, A. Shanmugasundaram, and D.-W. Lee, ‘Piezoresistive sensor-integrated PDMS cantilever: A new class of device for measuring the drug-induced changes in the mechanical activity of cardiomyocytes’, *Sens. Actuators B Chem.*, vol. 240, pp. 566–572, Mar. 2017, doi: 10.1016/j.snb.2016.08.167.

[18] F. Yu *et al.*, ‘Design, fabrication, and characterization of polymer-based cantilever probes for atomic force microscopes’, *J. Vac. Sci. Technol. B*, vol. 34, no. 6, p. 06KI01, Aug. 2016, doi: 10.1116/1.4960726.

[19] G. Genolet, M. Despont, P. Vettiger, D. Anselmetti, and N. F. de Rooij, ‘All-photoplastic, soft cantilever cassette probe for scanning force microscopy’, *J. Vac. Sci. Technol. B Microelectron. Nanometer Struct. Process. Meas. Phenom.*, vol. 18, no. 2, pp. 617–620, Mar. 2000, doi: 10.1116/1.591248.

[20] L. Chièze *et al.*, ‘Quantitative characterization of single-cell adhesion properties by atomic force microscopy using protein-functionalized microbeads’, *J. Mol. Recognit.*, vol. 32, no. 3, p. e2767, 2019, doi: 10.1002/jmr.2767.

[21] O. Thomas - - Chemin *et al.*, ‘Automated Bio-AFM Generation of Large Mechanome Data Set and Their Analysis by Machine Learning to Classify Cancerous Cell Lines’, *ACS Appl. Mater. Interfaces*, vol. 16, no. 34, pp. 44504–44517, Aug. 2024, doi: 10.1021/acsami.4c09218.

[22] W. A. Ducker, T. J. Senden, and R. M. Pashley, ‘Direct measurement of colloidal forces using an atomic force microscope’, *Nature*, vol. 353, no. 6341, pp. 239–241, Sep. 1991, doi: 10.1038/353239a0.

[23] N. Hosseini, M. Neuenschwander, O. Peric, S. H. Andany, J. D. Adams, and G. E. Fantner, ‘Integration of sharp silicon nitride tips into high-speed SU8 cantilevers in a batch fabrication process’, *Beilstein J. Nanotechnol.*, vol. 10, no. 1, pp. 2357–2363, Nov. 2019, doi: 10.3762/bjnano.10.226.

[24] V. Dremov, V. Fedoseev, P. Fedorov, and A. Grebenko, ‘Fast and reliable method of conductive carbon nanotube-probe fabrication for scanning probe microscopy’, *Rev. Sci. Instrum.*, vol. 86, no. 5, p. 053703, May 2015, doi: 10.1063/1.4921323.

[25] A. Savenko *et al.*, ‘Ultra-high aspect ratio replaceable AFM tips using deformation-suppressed focused ion beam milling’, *Nanotechnology*, vol. 24, no. 46, p. 465701, Nov. 2013, doi: 10.1088/0957-4484/24/46/465701.

[26] D. Caballero, G. Villanueva, J. A. Plaza, C. A. Mills, J. Samitier, and A. Errachid, ‘Sharp High-Aspect-Ratio AFM Tips Fabricated by a Combination of Deep Reactive Ion Etching and Focused Ion Beam Techniques’, *J. Nanosci. Nanotechnol.*, vol. 10, no. 1, pp. 497–501, Jan. 2010, doi: 10.1166/jnn.2010.1737.

[27] M. Y. Ali, W. Hung, and F. Yongqi, ‘A review of focused ion beam sputtering’, *Int. J. Precis. Eng. Manuf.*, vol. 11, no. 1, pp. 157–170, Feb. 2010, doi: 10.1007/s12541-010-0019-y.

[28] F. Zenhausern, M. Adrian, B. ten Heggeler‐Bordier, F. Ardizzoni, and P. Descouts, ‘Enhanced imaging of biomolecules with electron beam deposited tips for scanning force microscopy’, *J. Appl. Phys.*, vol. 73, no. 11, pp. 7232–7237, Jun. 1993, doi: 10.1063/1.354010.

[29] M. Samaan, H. Ekinci, R. Dey, X. Zhu, D. Pushin, and bo Cui, ‘Fabrication of high aspect ratio atomic force microscope probes using focused ion beam milled etch mask’, *Microelectron. Eng.*, vol. 267–268, p. 111909, Nov. 2022, doi: 10.1016/j.mee.2022.111909.

[30] E. Gacka, B. Pruchnik, M. Tamulewicz-Szwajkowska, D. Badura, I. W. Rangelow, and T. Gotszalk, ‘Fabrication of focused ion beam-deposited nanowire probes for conductive atomic force microscopy’, *Measurement*, vol. 234, p. 114815, Jul. 2024, doi: 10.1016/j.measurement.2024.114815.

[31] J. S. Lee *et al.*, ‘Multifunctional hydrogel nano-probes for atomic force microscopy’, *Nat. Commun.*, vol. 7, no. 1, p. 11566, May 2016, doi: 10.1038/ncomms11566.

[32] Y. F. Dufrêne, D. Martínez-Martín, I. Medalsy, D. Alsteens, and D. J. Müller, ‘Multiparametric imaging of biological systems by force-distance curve–based AFM’, *Nat. Methods*, vol. 10, no. 9, pp. 847–854, Sep. 2013, doi: 10.1038/nmeth.2602.

[33] A. M. Joshua, G. Cheng, and E. V. Lau, ‘Soft matter analysis via atomic force microscopy (AFM): A review’, *Appl. Surf. Sci. Adv.*, vol. 17, p. 100448, Oct. 2023, doi: 10.1016/j.apsadv.2023.100448.

[34] N. Alsharif, A. Burkatovsky, C. Lissandrello, K. M. Jones, A. E. White, and K. A. Brown, ‘Design and Realization of 3D Printed AFM Probes’, *Small*, vol. 14, no. 19, p. 1800162, 2018, doi: 10.1002/smll.201800162.

[35] R. C. L. N. Kramer *et al.*, ‘Multiscale 3D-printing of microfluidic AFM cantilevers’, *Lab. Chip*, vol. 20, no. 2, pp. 311–319, 2020, doi: 10.1039/C9LC00668K.

[36] G. Göring *et al.*, ‘Tailored probes for atomic force microscopy fabricated by two-photon polymerization’, *Appl. Phys. Lett.*, vol. 109, no. 6, p. 063101, Aug. 2016, doi: 10.1063/1.4960386.

[37] S. LoTurco, R. Osellame, R. Ramponi, and K. C. Vishnubhatla, ‘Hybrid chemical etching of femtosecond laser irradiated structures for engineered microfluidic devices’, *J. Micromechanics Microengineering*, vol. 23, no. 8, p. 085002, Jun. 2013, doi: 10.1088/0960-1317/23/8/085002.

[38] S. Kim, J. Kim, Y.-H. Joung, S. Ahn, J. Choi, and C. Koo, ‘Optimization of selective laser-induced etching (SLE) for fabrication of 3D glass microfluidic device with multi-layer micro channels’, *Micro Nano Syst. Lett.*, vol. 7, no. 1, p. 15, Oct. 2019, doi: 10.1186/s40486-019-0094-5.

[39] J. Gottmann, M. Hermans, N. Repiev, and J. Ortmann, ‘Selective Laser-Induced Etching of 3D Precision Quartz Glass Components for Microfluidic Applications—Up-Scaling of Complexity and Speed’, *Micromachines*, vol. 8, no. 4, Art. no. 4, Apr. 2017, doi: 10.3390/mi8040110.

[40] Y. Bellouard, ‘Shape memory alloys for microsystems: A review from a material research perspective’, *Mater. Sci. Eng. A*, vol. 481–482, pp. 582–589, May 2008, doi: 10.1016/j.msea.2007.02.166.

[41] J. Gottmann, ‘Microcutting and Hollow 3D Microstructures in Glasses by In-volume Selective Laser-induced Etching (ISLE)’, *J. Laser MicroNanoengineering*, vol. 8, no. 1, pp. 15–18, Feb. 2013, doi: 10.2961/jlmn.2013.01.0004.

[42] A. Butkutė *et al.*, ‘Optimization of selective laser etching (SLE) for glass micromechanical structure fabrication’, *Opt. Express*, vol. 29, no. 15, pp. 23487–23499, Jul. 2021, doi: 10.1364/OE.430623.

[43] ‘All‐Glass 3D Optofluidic Microchip with Built‐in Tunable Microlens Fabricated by Femtosecond Laser‐Assisted Etching - Hu - 2018 - Advanced Optical Materials - Wiley Online Library’. Accessed: Jul. 16, 2025. [Online]. Available: https://advanced-onlinelibrary-wiley-com.gorgone.univ-toulouse.fr/doi/full/10.1002/adom.201701299

[44] L. Beckmann *et al.*, ‘Selective laser-induced etching for novel 3D microphotonic devices’, in *Laser-based Micro- and Nanoprocessing XVIII*, SPIE, Mar. 2024, pp. 7–14. doi: 10.1117/12.3002335.

[45] L. Jonušauskas, D. Mackevičiūtė, G. Kontenis, and V. Purlys, ‘Femtosecond lasers: the ultimate tool for high-precision 3D manufacturing’, *Adv. Opt. Technol.*, vol. 8, no. 3–4, pp. 241–251, Jun. 2019, doi: 10.1515/aot-2019-0012.

[46] A. Butkutė and L. Jonušauskas, ‘3D Manufacturing of Glass Microstructures Using Femtosecond Laser’, *Micromachines*, vol. 12, no. 5, Art. no. 5, May 2021, doi: 10.3390/mi12050499.

[47] C. Hnatovsky *et al.*, ‘Pulse duration dependence of femtosecond-laser-fabricated nanogratings in fused silica’, *Appl. Phys. Lett.*, vol. 87, no. 1, p. 014104, Jul. 2005, doi: 10.1063/1.1991991.

[48] C. A. Ross, D. G. MacLachlan, D. Choudhury, and R. R. Thomson, ‘Optimisation of ultrafast laser assisted etching in fused silica’, *Opt. Express*, vol. 26, no. 19, p. 24343, Sep. 2018, doi: 10.1364/OE.26.024343.

[49] I. H. Jafri, H. Busta, and S. T. Walsh, ‘Critical point drying and cleaning for MEMS technology’, in *MEMS Reliability for Critical and Space Applications*, SPIE, Aug. 1999, pp. 51–58. doi: 10.1117/12.359371.

[50] P. S. Timashev, S. L. Kotova, N. N. Glagolev, N. A. Aksenova, A. B. Solovieva, and V. N. Bagratashvili, ‘Cleaning of cantilevers for atomic force microscopy in supercritical carbon dioxide’, *Russ. J. Phys. Chem. B*, vol. 8, no. 8, pp. 1081–1086, Dec. 2014, doi: 10.1134/S1990793114080168.

[51] T. R. Albrecht, P. Grütter, D. Horne, and D. Rugar, ‘Frequency modulation detection using high‐Q cantilevers for enhanced force microscope sensitivity’, *J. Appl. Phys.*, vol. 69, no. 2, pp. 668–673, Jan. 1991, doi: 10.1063/1.347347.

[52] R. Garcı́a and R. Pérez, ‘Dynamic atomic force microscopy methods’, *Surf. Sci. Rep.*, vol. 47, no. 6, pp. 197–301, Sep. 2002, doi: 10.1016/S0167-5729(02)00077-8.

[53] J. S. Villarrubia, ‘Algorithms for scanned probe microscope image simulation, surface reconstruction, and tip estimation’, *J. Res. Natl. Inst. Stand. Technol.*, vol. 102, no. 4, p. 425, Jul. 1997, doi: 10.6028/jres.102.030.

[54] G. Schürmann *et al.*, ‘Fabrication and characterization of a silicon cantilever probe with an integrated quartz-glass (fused-silica) tip for scanning near-field optical microscopy’, *Appl. Opt.*, vol. 40, no. 28, pp. 5040–5045, Oct. 2001, doi: 10.1364/AO.40.005040.
